# The VP1 Protein of Porcine Teschovirus Inhibits the Innate Immune Response to Viral Infection by Blocking MDA5 Activation

**DOI:** 10.1155/2024/6649669

**Published:** 2024-01-08

**Authors:** Yuying Li, Xinyu Zhang, Baopeng Zhao, Chenchen Zhao, Xiaoxiao Lei, Haixin Huang, Chengkai Li, Min Zheng, Tian Lan, Wenchao Sun, HuiJun Lu

**Affiliations:** ^1^College of Animal Sciences, Institute of Preventive Veterinary Medicine, Zhejiang University, Hangzhou, Zhejiang 310000, China; ^2^Institute of Virology, Wenzhou University, Wenzhou, Zhejiang 325035, China; ^3^Guangxi Center for Disease Prevention and Control, Nanning, Guangxi 530001, China; ^4^Changchun Veterinary Research Institute, Jilin Academy of Agricultural Sciences, Changchun, Jilin 130000, China

## Abstract

Porcine teschovirus (PTV) can cause reproductive dysfunction and respiratory diseases, with high mortality rates for sick pigs. Among Picornaviridae family virus-encoded proteins, the VP1 structural protein is critical for viral immune evasion. However, whether PTV VP1 inhibits the type I interferon (IFN) response remains unknown. Here, it shows that the PTV VP1 protein significantly hinders the activation of NF-*κ*B, impairing Sendai virus-induced expression of beta IFN (IFN-*β*). Further studies revealed that VP1 targets and interacts with the MDA5 factor in the RIG-I like receptors pathway. More importantly, the VP1 protein interacts with the caspase activation and recruitment domain and Hel domains of MDA5, blocking IFN-*β* expression. Our findings provide evidence about the VP1 protein of PTV hinders MDA5 activation and may represent a viral mechanism to escape the innate immune response.

## 1. Introduction

Porcine teschovirus (PTV) is classified within the *Teschovirus* genus belonging to the *Picornaviridae* family [1] and is characterized as a nonenveloped, icosahedral RNA virus with a positive-strand polarity [2, 3]. PTV can cause reproductive dysfunction and respiratory diseases in nearly all infected pigs. PTV first emerged in the Czech Republic in 1929 [4], quickly spreading to Australia, Africa, Europe, and North America, with high mortality rates among infected pigs [5, 6]. Teschovirus currently includes 19 genotypes and 13 serotypes [7]. PTV1, 2, 3, 5, and 11 are all capable of causing poliomyelitis, with PTV1 exhibiting the highest pathogenicity and mortality rate [8–10]. PTV-2 demonstrates a preference for infecting the nervous system and predominantly causes mild hind-limb paralysis in pigs. Lesions primarily affect the central nervous system, encompassing the spinal cord, brainstem, and cerebellum, while its presence in the cerebrum is comparatively restricted [11].

PTV consists of an Open Reading Frame, including structural proteins (VP1, VP2, VP3, and VP4) and nonstructural proteins (L, 2A, 2B, 2C, 3A, 3B, 3C, and 3D) [12–14]. The PTV VP1 protein is a nucleocapsid protein that exhibits species-specific characteristics [1]. VP1A107, positioned at residue 107 of the capsid protein, serves as a crucial element in the exceedingly efficient replication of enterovirus 71 (EV71) [15]. This particular mutation significantly enhances EV71's replicative capacity and is a key determinant of viral pathogenesis. VP1–145 is involved in the cell-specific virus replication and pathogenesis of EV71-infected individuals in vivo [16]. Additionally, previous research has indicated that the S154D mutation in VP1 has significant effects on the replication and pathogenicity of type Asia1 foot-and-mouth disease virus (FMDV) [17]. The inhibition of type I interferon (IFN-I) by FMDV VP1 is attributed to its interaction with sorcin, a soluble calcium-binding protein known to govern cellular response to viral infections [18]. FMDV VP1 can effectively inhibit viral replication by suppressing GBP1, which is an IFN-stimulated gene [19]. While the VP1 protein plays a vital role in *Picornaviridae* family virus infections, the mechanism of PTV VP1 evades the host's innate immune response remains unclear.

IFN is the main defense system of the host, which response to viral invasion [20, 21]. RIG-I like receptors (RLRs), a class of RNA-dependent ATPases, play a crucial role in this process. These ATP hydrolyzing enzymes contain highly conserved DExD/H structures. The identification and recognition of a wide range of RNA viruses are accomplished by two primary types of RLRs, namely RIG-I (retinoic acid-inducible gene I) and MDA5 (melanoma differentiation factors) [22]. After detecting viral RNA, the RIG-I/MDA5 receptors trigger MAVS-dependent antiviral signaling and interact with the essential adaptor protein MAVS [23]. This interaction triggers the activation of the IKK*α*-IKK*β*-IKK*γ* trimeric complex, resulting in the activation of NF-*κ*B [24]. Additionally, it activates the IKK*ε*-TBK1 complex, leading to the phosphorylation and dimerization of interferon transcription factors IRF3/IRF7 [25]. Activated IRF3/IRF7 and NF-*κ*B translocate into the nucleus and bind to the coactivator CBP (CREB-binding protein), forming a transcription enhancer that promotes the transcription of IFN-*β*, thus establishing the antiviral state of the cell [26].

In this study, we discovered the PTV VP1 protein directly targeted MDA5 through its 2CARD and Hel domains to effectively inhibit IFN-*β* production and block NF-*κ*B activation. To our knowledge, this is the first report of the underlying mechanism by which MDA5 suppresses type I IFN production, which is associated with PTV.

## 2. Materials and Methods

### 2.1. Cell Culture and Viruses

ST and HEK293T cells were cultured in Dulbecco's modified Eagle's medium at 37°C. Stocks of PTV type 2 strain GX/2020, Sendai virus (SeV), and recombinant vesicular stomatitis virus (VSV–GFP) were maintained in our laboratory.

### 2.2. Antibodies and Reagents

Total RNA Extractor Kit (Sangon Biotech, No. B511311), PrimeScript™ RT-PCR Kit (TaKaRa, RR014A). Anti-Flag (bsm-33346M) and anti-HA (bsm-33003M) mouse monoclonal antibodies, NF-*κ*B polyclonal antibody (Proteintech, 10745-1-AP), and phospho-NF-*κ*B monoclonal antibodies (Cell Signaling Technology, #3033) were used for western blotting (WB). Double-Luciferase Reporter Assay Kit (TransGen, FR201-02) and Exfect transfection reagent (Vazyme Biotechnology, T101-01/02) were used.

### 2.3. Plasmids Construction

IFN-*β*, NF-*κ*B promoter luciferase reporter plasmids, and mammalian expression plasmids (RIG-I, MDA5, MAVS, and TBK1) were stored in our laboratory. VP1 was cloned into the pXJ-HA vector. Three characteristic functional domains of MDA5—the 2CARD domain (aa 1–285), the helicase domain (aa 286–874), and the C-terminal domain (CTD) (aa 875–1024) [27] were cloned into the pCAGGS-Flag using the primers listed in Table 1, and the resulting expression constructs were named 2CARD (MDA5), Hel (MDA5), and CTD (MDA5), respectively.

### 2.4. RNA Extraction and qPCR

The RNA Extractor kit (Sangon Biotech) was employed to extract total cellular mRNA. To synthesize cDNA, 500 ng of RNA was reverse transcribed using the PrimeScript™ RT-PCR Kit (TaKaRa, RR014A). qPCR using the GoTaq® qPCR Master enzyme premix (Promega, A600A) for SYBR fluorescence detection. Each set included three replicate wells, and the experiment was conducted in triplicate. The primers used for qPCR can be found in Table 1. The expression of the target gene was compared to the endogenous GAPDH level, and the relative expression was determined using the 2^−*ΔΔ*CT^ method.

### 2.5. Luciferase Reporter Assay

IFN-*β*-Luc or NF-*κ*B-luc luciferin reporter plasmid (500 ng/well) and control plasmid pRL-TK (10 ng/well) were cotransfected into 293 T cells in 12-well plates using ExFect Transfection Reagent (Vazyme, China). After introducing the plasmids into the cells, they were thereafter exposed to stimulation, either with or without SeV for 12 hr. The Dual Luciferase Reporter System (TransGen, China) was used to detect luciferase activity, with normalization to the values of the firefly fluorescence and sea kidney fluorescence. All the data of the reporter gene assay were from three separate experiments.

### 2.6. Coimmunoprecipitation (Co-IP)

Cell lysis was performed using NP-40 lysis buffer at 4°C supplemented with phenylmethylsulfonyl fluoride. Following centrifugation at 12,000 × *g* for 10 min, the supernatant was combined with specific antibodies and Pierce™ Protein A/G (ThermoScientific™, 88802) and incubated overnight. Lastly, the eluted beads were added to the loading buffer for WB analysis after boiling.

### 2.7. WB

The protein samples were separated on sodium dodecyl sulfate–polyacrylamide gel electrophoresis gel and transferred to the polyvinylidene fluoride membrane. After blocking with 5% skim milk, it was incubated with the primary antibody at 4°C overnight. After washing, the membrane was incubated with the horseradish peroxidase-conjugated secondary antibody for 1 hr. Finally, the protein bands were observed by chemiluminescence and photographed using the ChemiDoc™ XRS + System.

### 2.8. Indirect Immunofluorescence Assay (IFA)

ST cells were seeded onto a glass culture dish and subsequently exposed to SeV infection for 12 hr the next day. The cells were fixed with 4% paraformaldehyde for 20 min and permeabilized with 0.5% Triton X-100 for 10 min. Then, it was blocked with 5% skim milk and incubated with proper primary antibodies at 4°C overnight. The cells were incubated with the secondary antibodies for 1 hr. The nuclei of the cells were stained with DAPI. Cells were imaged using a confocal microscope (FV3000, Olympus, Japan).

### 2.9. Statistics

All data are presented as the means ± SDs from three independent experiments. Repeated measures one-way analysis of variance was used for statistical significance analysis. GAPDH expression served as a loading control. Meaning symbol is defined as follows: NS, no significant;  ^*∗*^*P*  < 0.05,  ^*∗∗*^*P*  < 0.01,  ^*∗∗∗*^*P*  < 0.001.

## 3. Results

### 3.1. The Viral Protein VP1 Is a Negative Regulator of IFN-*β* Production

**Figure 1:**
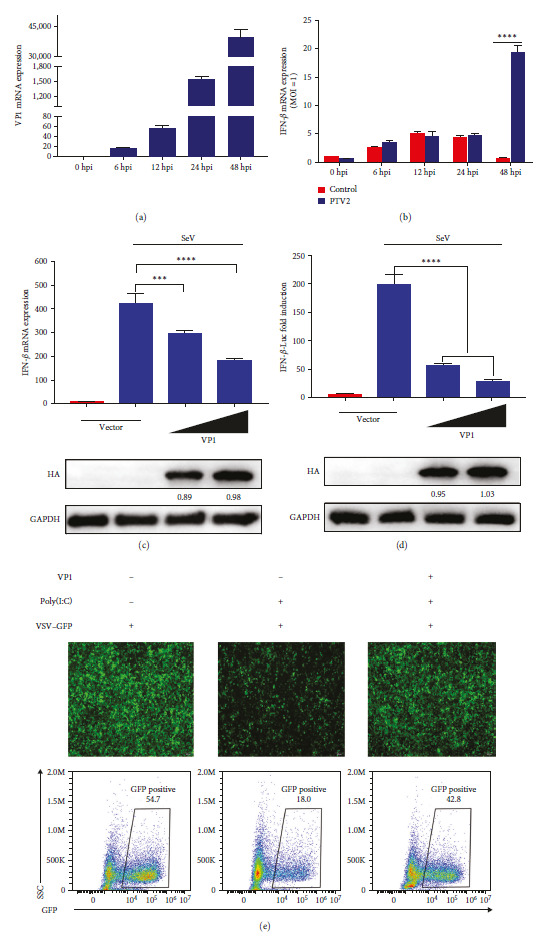
The PTV VP1 protein curbs SeV-mediated IFN-*β* activation: (a and b) reproduction of PTV in ST cells was examined by infecting the cells with PTV at a MOI of 1 for specified durations: (a) the expression levels of VP1 mRNA were quantified using qPCR at different time points (0, 6, 12, 24, and 48 hr) postinfection, (b) the levels of IFN-*β* mRNA expression were also measured by qPCR at the same time points; (c) HEK293T cells were introduced to VP1 plasmids (500 ng, 1,000 ng) via transfection 24 hr, then infected with SeV for 12 hr. The levels of IFN-*β* mRNA expression were again measured by qPCR; (d) HEK293T cells were cotransfected with the IFN-*β*-Luc, pRL-TK, and VP1 plasmids (500 ng, 1,000 ng) and then subjected to SeV infection. The luminescent signal was assessed after a 12-hr period utilizing a dual-luciferase detection method; (e) HEK293T cells transfected with an empty vector and a vector encoding VP1 protein for 24 hr, then infected with VSV–GFP and poly(I:C) for 12 hr.

During ST cell infection, we observed that PTV induces a low level of IFN-*β* production (Figures 1(a) and 1(b)). Research has shown that the VP1 protein, a component of PTV capsid protein, can enhance virus replication [17]. Therefore, there may be an internal regulatory mechanism to inhibit IFN-*β* production while promoting virus replication. Our objective is to elucidate the molecular pathway by which VP1 enhances PTV replication. To achieve this, we employed qPCR to assess the influence of VP1 on the production of IFN-*β*, which is caused by the RNA virus SeV from the Paramyxoviridae family. Our results showed that VP1 protein inhibited SeV-induced IFN-*β* production (Figure 1(c)).

Additionally, the dual luciferase reporter gene was employed to investigate the impact of VP1 on the activity of the SeV-activated IFN-*β* promoter. The findings indicated a dose-dependent inhibition of IFN-*β* promoter activation by VP1 (Figure 1(d)). Moreover, viral replication can be hindered by interferon. Hence, to evaluate viral replication, experiments were conducted using the vesicular stomatitis virus (VSV–GFP) and fluorescence intensity as a measurable parameter. Results from the VSV experiments indicated a reduction in GFP fluorescence in cells stimulated with poly(I:C) and producing IFN-*β*, affirming the inhibition of VSV–GFP proliferation. However, the partial restoration of GFP fluorescence was observed upon the overexpression of VP1, indicating a partial recovery in VSV–GFP proliferation (Figure 1(e)). Summarily, VP1 displays inhibitory effects on IFN-*β* production.

### 3.2. VP1 Blocks IFN-*β* Production by Inhibiting NF-*κ*B Activation

**Figure 2:**
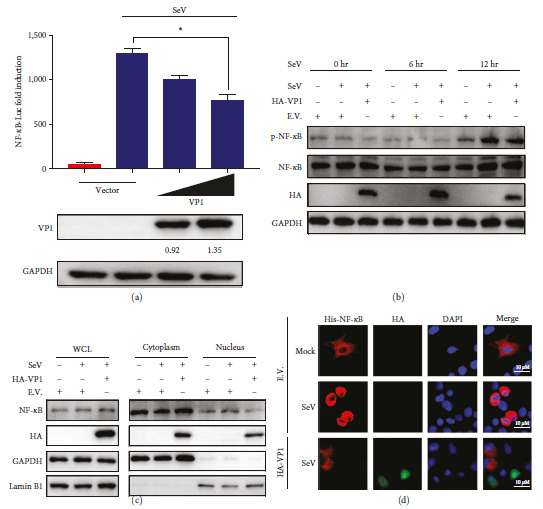
The PTV VP1 protein inhibits NF-*κ*B activation: (a) HEK293T cells underwent co-transfection with 500 ng and 1,000 ng of the NF-*κ*B reporter, pRL-TK, and VP1 plasmids, respectively. Following transfection, the cells were either infected with SeV. After 12 hr incubation, luciferase activity was quantified using a dual-luciferase assay; (b and c) ST cells were subjected to transfection with the VP1 expression plasmid and an empty vector 24 hr, followed by infection with SeV for 0, 6, and 12 hr. The collected cells were subjected to Western blot to assess the protein levels of p-NF-*κ*B and NF-*κ*B: (b) following a 12 hr infection with SeV, the abundance of NF-*κ*B in the cytoplasmic and nuclear fractions was assessed using western blot analysis, (c) GAPDH and LaminB1 as markers for cytoplasmic and nuclear fractions, respectively. Whole-cell lysate—WCL; (d) ST cells transfected with the plasmids as indicated for 24 hr, then infected with SeV for 12 hr. The subcellular localizations of His-NF-*κ*B (red), HA-VP1 (green), and the nuclear marker DAPI (blue) were determined by immunofluorescence staining.

To determine the inhibitory influence of the VP1 protein on the activation of NF-*κ*B mediated IFN-I signaling, we performed an NF-*κ*B reporter assay to assess the influence of VP1 on NF-*κ*B activation. Overexpression of VP1 suppressed the activation of the NF-*κ*B promoter induced by SeV (Figure 2(a)). NF-*κ*B activation is characterized by phosphorylation and nuclear translocation, and the effects of VP1 were investigated by immunoblot analyses. The findings demonstrated that stimulation with SeV resulted in a substantial rise in phosphorylation of NF-*κ*B, whereas the overexpression of VP1 protein effectively inhibited the NF-*κ*B phosphorylation induced by SeV (Figure 2(b)). Moreover, VP1 significantly hindered the SeV-induced nuclear translocation of NF-*κ*B, which consequently led to a decrease in the translocation of NF-*κ*B into the nucleus (Figure 2(c)). The nuclear entry of NF-*κ*B caused by SeV was curbed by VP1 overexpression, as demonstrated by the IFA results (Figure 2(d)). These findings indicate that VP1 restrains SeV-induced NF-*κ*B activation, ultimately decreasing IFN-*β* production.

### 3.3. The VP1 Protein Targets MDA5

**Figure 3:**
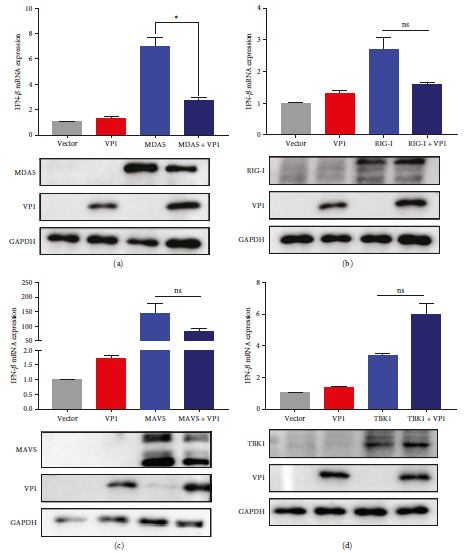
The PTV VP1 protein targets MDA5 and suppresses IFN-*β* production: (a–d) effects of VP1 on components of the RLR pathway. ST cells were transfected with various plasmids, including RIG-1, MDA5, MAVS, or TBK1, along with a control vector or a VP1-expressing plasmid. After 24 hr, the IFN-*β* activity was quantified using qPCR.

To gain deeper insights into the molecular mechanism behind the VP1-mediated suppression of NF-*κ*B-dependent IFN-*β* production, further investigations were conducted; we conducted experiments to evaluate the impact of VP1 on the activation of IFN-*β* caused by different components of the RLR pathway, namely RIG-1, MDA5, MAVS, and TBK1. Subsequent analysis using qPCR demonstrated a significant reduction in MDA5-triggered IFN-*β* activation upon VP1 treatment. Conversely, no substantial inhibitory effect was observed when IFN-*β* activation was induced by RIG-1, MAVS, or TBK1 (Figure 3(a)–3(d)). These findings strongly suggest that the VP1 protein specifically targets the MDA5 protein.

### 3.4. VP1 Inhibits MDA5 Function by Binding to MDA5

**Figure 4:**
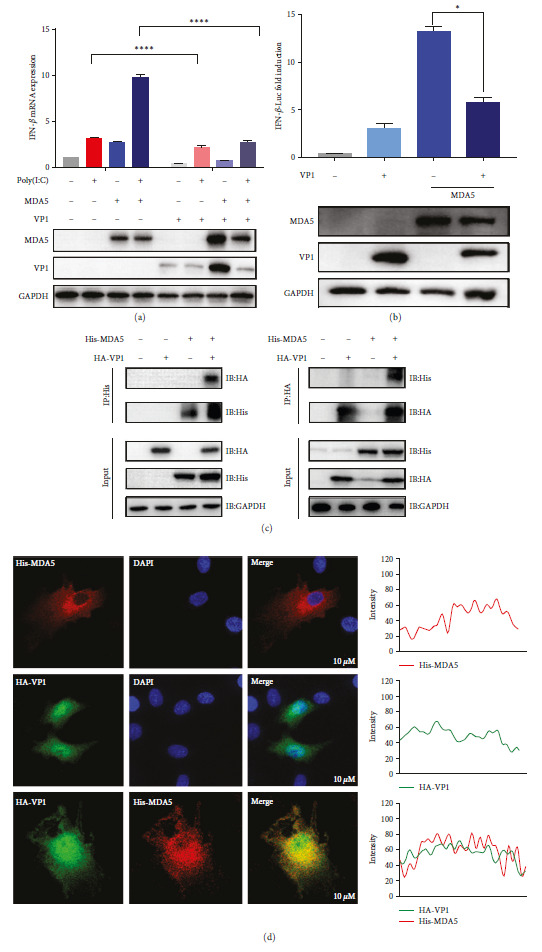
The PTV VP1 inhibits MDA5 function by directly binding to MDA5: (a) the capacity of VP1 to hinder MDA5-triggered IFN-*β* production was evaluated by transfecting ST cells with MDA5 and a vector expressing VP1, subsequently exposed to poly(I:C) treatment duration of 12 hr; (b) The impact of VP1 on the activation of the IFN-*β* promoter mediated by MDA5 was studied in HEK293T cells transfected with the IFN-*β* reporter, pRL-TK, and MDA5 along with a control vector or a VP1-expressing vector, followed by measurement of IFN-*β* activities employing a dual-luciferase reporter-based assay after 36 hr; (c) The Co-IP assay was performed to validate the interaction between VP1 and MDA5 in HEK293T cells. This involved cotransfecting His-tagged MDA5 and HA-VP1 into the cells. After 36 hr, the cell lysates were utilized for immunoprecipitation employing either anti-His or anti-HA mouse monoclonal antibodies, followed by immunoblotting using anti-His, anti-HA, and anti-GAPDH mouse monoclonal antibodies; (d) colocalization of MDA5 with VP1 was observed in ST cells through confocal microscopy analysis. Representative images of His-MDA5 colocalization with HA-VP1 were captured, and the intensity of each band was quantified using ImageJ software and depicted utilizing GraphPad Prism 9.0.

To confirm that VP1 could inhibit IFN-*β* activation by targeting MDA5, we employed qPCR to examine the impact of VP1 and MDA5 on the levels of IFN-*β* activation caused by poly(I:C) stimulation. MDA5 stimulates the production of interferon. The VP1 protein inhibits interferon activity. Poly(I:C) treatment significantly increased MDA5-stimulated interferon, while VP1 protein inhibited this synergistic effect (Figure 4(a)). Furthermore, experiments using luciferase revealed that VP1 effectively suppressed the initiation of IFN-*β* promoter activation by MDA5 (Figure 4(b)).

Thus, we hypothesized that VP1 targets MDA5. The Co-IP assay demonstrated a strong association between VP1 and MDA5 (Figure 4(c)). We performed an immunofluorescence staining assay to confirm the interaction between VP1 and MDA5. We observed colocalization between the viral protein VP1 and MDA5 (Figure 4(d)).

### 3.5. VP1 Inhibits MDA5 Function by Binding of the 2CARD and Hel Domains to MDA5

**Figure 5:**
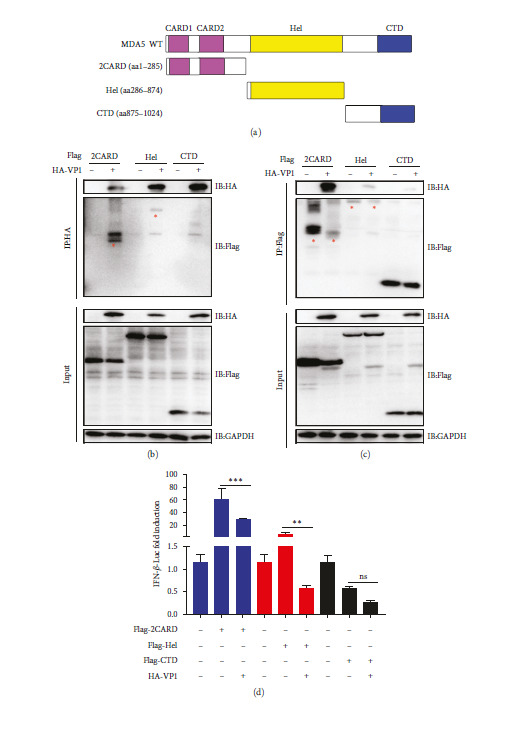
The PTV VP1 inhibits MDA5 function by direct binding of the 2CARD and Hel domain to MDA5: (a) a diagrammatic illustration of the distinct domains of MDA5 is displayed; (b and c) the MDA5 domain that interacts with VP1 is identified through CO-IP assays. HEK293T cells were simultaneously transfected with HA-tagged VP1 and constructs encoding either Flag-tagged 2CARD, Hel, or CTD, together with an empty vector. After transfection, the lysates were analyzed by immunoblotting using antibodies specific to either Flag or HA tags; (d) the inhibitory effects of VP1 on the induction of IFN-*β* facilitated by 2CARD, Hel, or CTD were assessed. HEK293T cells were subjected to transfection with the IFN-*β*-Luc and pRL-TK, along with a control vector or a VP1, 2CARD, Hel, and CTD plasmids. Luciferase signals were quantified employing a dual-luciferase reporter assay.

We investigated whether VP1 directly binds to the MDA5 receptor at the site where the RNA ligand binds, thereby modulating MDA5 function. MDA5 comprises three domains: two N-terminal CARD domains, a helicase domain (Hel), and a CTD [28] (Figure 5(a)). Domain mapping studies revealed that VP1 interplays with the 2CARD and Hel domains of MDA5 (Figures 5(b) and 5(c)). Then, we measured the activation of the IFN-*β* promoter driven by 2CARD, Hel, and the CTD to investigate whether VP1 negatively regulated IFN-*β* promoter activity through the 2CARD and Hel domains of MDA5. The results demonstrated a significant decrease in IFN-*β* promoter activation driven by 2CARD and Hel in the presence of VP1 (Figure 5(d)), suggesting that VP1 exerts a critical affect in inhibiting the induction of IFN-*β* mediated by the 2CARD and Hel domains of MDA5.

## 4. Discussion

PTV is the pathogen responsible for poliomyelitis, reproductive disorders, encephalomyelitis, and gastrointestinal diseases in swine. In recent years, PTV has affected a substantial number of pig farms in China, highlighting the importance of understanding and preventing PTV infection [29]. In 2003, PTV1 was first isolated in China, and then the disease was continuously detected [30]. In 2009, emerging evidence indicates that robust purifying selection and recombination are crucial factors influencing the genetic diversity of PTV. In 2010, a PTV strain isolated in Heilongjiang Province showed recombination, indicating that the recombination of the virus may promote the evolution of PTV [10]. In 2011, at least two independent recombination events occurred in Europe and China's PTV-8 strains during its evolution [31]. In 2017, nine recombinations were detected in PTV-HuN in Hunan in 2017 [32]. In 2018, PTV2 infection was shown to impair autophagy to hinder its further replication in PK-15 cells [33], but studies on PTV pathogenesis are lacking.

The VP1 protein is a crucial structural element of the Picornaviridae family viruses and plays a vital role in the viral capsid. Recent research has demonstrated that the modification of amino acids in the FMDV VP1 protein can significantly increase viral infectivity and pathogenicity [17]. VP1 can also affect the stability and pathogenicity of the virus. The species specificity of the PTV VP1 protein and its mechanism for evading host innate immune responses are still unknown. Therefore, this research with the purpose of explore the pathogenicity of the PTV VP1 structural protein and its possible role in impeding IFN production. In this study, PTV VP1 suppressed SeV-triggered IFN-*β* activation (Figure 1(b)), indicating that PTV VP1 hinders IFN-*β* activation. NF-*κ*B is a necessary transcription factor involved in the type I IFN signaling pathway. As a result, our research aimed to investigate the influence of VP1 on NF-*κ*B activity. Our findings demonstrated that VP1 greatly hindered NF-*κ*B phosphorylation and nuclear translocation, bring about the disruption of NF-*κ*B activation and the prevention of IFN-*β* production (Figure 2).

MDA5 is a member of the RIG-I-like helicase family and exerts a critical affect in the detection of viral RNA in the cytoplasm. MDA5 has helicase regions, including helicase structure 1 (Hel-1), helicase structure 2 (Hel-2), and a specific intermediate insertion region (Hel-2i). The terminal region also contains a CTD [34]. The Hel and CTD regions play a crucial role in binding specific RNA ligands [35]. Additionally, the N-terminus of MDA5 contains two recruitment activation domains, known as caspase activation and recruitment domains (CARDs), which facilitate signal transduction by engaging with downstream adaptor proteins. Upon activation, MDA5 stimulates the synthesis of type I IFN, thereby bolstering the host's immune response against viral infections [36]. In general, negative-strand RNA viruses, including the influenza virus [37] and Bunya virus [38], as well as the positive-strand Japanese encephalitis virus [39], are known to trigger the activation of RIG-I. Further, small positive-strand RNA viruses, like the viruses belonging to the *Picornaviridae* family [40] and hepatitis D virus [41], typically elicit the activation of MDA5.

**Figure 6:**
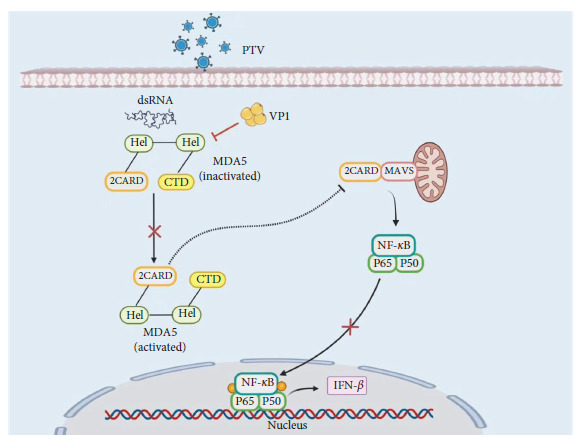
Model depicting PTV structural protein VP1-mediated viral suppression by blocking MDA5 and NF-*κ*B activation during PTV infection. VP1 exhibits the ability to suppress the expression of IFN-*β* by obstructing the activation of NF-*κ*B. The binding domain localization demonstrates the interaction between VP1 and the CARD and Hel domains of MDA5, potentially reducing its RNA ligand-binding capability. Pointed arrows represent activation, while blunt-ended lines indicate inhibition (created using https://www.biorender.com).

In our study, we found that the VP1 protein targets MDA5 and interacts with its CARD and Hel domains, effectively inhibiting IFN-*β* production. Specifically, the Hel and CTD domains in MDA5 are RNA-binding regions. CTD recognizes RNA as the first step in the receptor activation process and subsequently binds to Hel. ATP hydrolysis releases energy to promote further changes in the spatial structure of MDA5, which subsequently releases the CARD region, interacts with the downstream key signal molecule MAVS, promotes signal transduction, and induces type 1 IFN production [42]. Therefore, the VP1 protein may compete with RNA for binding to MDA5, resulting in the inhibition of MDA5-mediated IFN-*β* production. Furthermore, the VP1 protein interferes with the Hel regions of MDA5, which may reduce its binding ability to dsRNA. The binding to CARD hinders the release of the CARD region after MDA5 activation, preventing its interaction with the downstream key signaling molecule MAVS and thereby inhibiting the production of IFN (Figure 6).

In summary, we demonstrated that PTV VP1 exerts an affect on the regulation of the host's immune response to type I IFN. Furthermore, our study reveals that VP1 interacts with MDA5 via its 2CARD and Hel domains, resulting in the inhibition of the downstream NF-*κ*B pathway. As a consequence, this leads to a reduction in host IFN signaling, promoting viral replication. These findings offer novel insights into the pathogenesis of PTV and the evasion strategies employed by VP1 to counteract the host's innate antiviral immune response.

## Figures and Tables

**Table 1:** Primers used for cloning and qPCR.

Primer name	Forward sequence (5′–3′)	Reverse sequence (5′–3′)
VP1	cccgattacgcctccggatccGGAAATGAAAGTTCACCTCTTCAAC	ggcggccgcctcgagaagcttTTGGAATGACATGGTAGTCATGGC
2CARD	gatgacgacgataaggaattcATGTCGTCGGATGGGTATTCCGCGG	attaagatctgctagctcgagGGTGCCTGAATCACTGCCCATGTTG
Hel	gatgacgacgataaggaattcATGGGAAGTGATTCAGATGAAGAGA	attaagatctgctagctcgagCTGTAATTCCAAAATCTTATGAGCA
CTD	gatgacgacgataaggaattcATGCAAAGTATAATGGAAAAGAAAA	attaagatctgctagctcgagTCAGTCCTCATCACTAGACAAACAA
P-IFN-*β*	CATCCTCCAAATCGCTCTCC	ACATGCCAAATTGCTGCTCC
p-RIG-I	CTGGAGCTTGCTTTACCTGC	CCTTCCCCTTTCGTCCTTGT
P-MDA5	GGAGTCAAAGCCCACCATCT	GCCACCGTGGTAGCGATAAG
P-MAVS	GGCATCAGAAGCAGGACACAGAAC	CAGTGGAGGAGGAGGCAGTAGAC
P-TBK1	AAGAGGAGACAACAACAAG	ACCCACCACATCTCTCAAA

## Data Availability

The original contributions presented in the study are included in the article/Supplementary Material. Further inquiries can be directed to the corresponding authors.

## Supplementary Materials

Supplementary Materials Figure S1: the statistics table of Figure 3. This data analysis primarily utilized Prism 9.0 software, specifically the RM one-way ANOVA feature. Figure S2: all western blot raw images.
