# Supplementary material for: The VP1 Protein of Porcine Teschovirus Inhibits the Innate Immune Response to Viral Infection by Blocking MDA5 Activation
Source: Transbound Emerg Dis. 2024 Jan 8;2024:6649669. doi: 10.1155/2024/6649669 (PMC12017125; doi:10.1155/2024/6649669)
Supplement: Supplementary Materials — Figure S1: the statistics table of Figure 3. This data analysis primarily utilized Prism 9.0 software, specifically the RM one-way ANOVA feature. Figure S2: all western blot raw images. [file 6649669.f1.docx]

Fig.S1. The statistics table of Fig.3. This data analysis primarily utilized Prism 9.0 software, specifically the RM one-way ANOVA feature.

| Tukey's multiple comparisons test | Mean Diff. | 95.00% CI of diff. | Below threshold? | Summary | Adjusted P Value | |
| --- | --- | --- | --- | --- | --- | --- |
| Vector vs. VP1 | -0.3287 | -0.5757 to -0.08172 | Yes | * | 0.0288 | A-B |
| Vector vs. MDA5 | -6.008 | -8.539 to -3.477 | Yes | ** | 0.0094 | A-C |
| Vector vs. MDA5+VP1 | -1.691 | -2.579 to -0.8041 | Yes | * | 0.0143 | A-D |
| VP1 vs. MDA5 | -5.679 | -8.457 to -2.902 | Yes | * | 0.0125 | B-C |
| VP1 vs. MDA5+VP1 | -1.363 | -2.119 to -0.6064 | Yes | * | 0.016 | B-D |
| MDA5 vs. MDA5+VP1 | 4.317 | 1.117 to 7.517 | Yes | * | 0.028 | C-D |
| Test details | Mean 1 | Mean 2 | Mean Diff. | SE of diff. | n1 | n2 |
| Vector vs. VP1 | 1 | 1.329 | -0.3287 | 0.03565 | 3 | 3 |
| Vector vs. MDA5 | 1 | 7.008 | -6.008 | 0.3653 | 3 | 3 |
| Vector vs. MDA5+VP1 | 1 | 2.691 | -1.691 | 0.128 | 3 | 3 |
| VP1 vs. MDA5 | 1.329 | 7.008 | -5.679 | 0.4009 | 3 | 3 |
| VP1 vs. MDA5+VP1 | 1.329 | 2.691 | -1.363 | 0.1091 | 3 | 3 |
| MDA5 vs. MDA5+VP1 | 7.008 | 2.691 | 4.317 | 0.4618 | 3 | 3 |

| Vector vs. VP1 | -0.3287 | -0.5757 to -0.08172 | Yes | * | 0.0288 | |
| --- | --- | --- | --- | --- | --- | --- |
| Vector vs. RIG-I | -1.725 | -3.128 to -0.3226 | Yes | * | 0.0335 | A-C |
| Vector vs. RIG-I+VP1 | -0.6001 | -0.8608 to -0.3395 | Yes | * | 0.01 | A-D |
| VP1 vs. RIG-I | -1.397 | -3.004 to 0.2106 | No | ns | 0.0653 | B-C |
| VP1 vs. RIG-I+VP1 | -0.2714 | -0.7420 to 0.1993 | No | ns | 0.1385 | B-D |
| RIG-I vs. RIG-I+VP1 | 1.125 | -0.01939 to 2.270 | No | ns | 0.0517 | C-D |
| Test details | Mean 1 | Mean 2 | Mean Diff. | SE of diff. | n1 | n2 |
| Vector vs. VP1 | 1 | 1.329 | -0.3287 | 0.03565 | 3 | 3 |
| Vector vs. RIG-I | 1 | 2.725 | -1.725 | 0.2024 | 3 | 3 |
| Vector vs. RIG-I+VP1 | 1 | 1.6 | -0.6001 | 0.03762 | 3 | 3 |
| VP1 vs. RIG-I | 1.329 | 2.725 | -1.397 | 0.2319 | 3 | 3 |
| VP1 vs. RIG-I+VP1 | 1.329 | 1.6 | -0.2714 | 0.06793 | 3 | 3 |
| RIG-Ivs.RIG-I+VP1 | 2.725 | 1.6 | 1.125 | 0.1652 | 3 | 3 |
| Vector vs. VP1 | -0.3287 | -0.5757 to -0.08172 | Yes | * | 0.0288 | A-B |

| Tukey's multiple comparisons test | Mean Diff. | 95.00% CI of diff. | Below threshold? | Summary | Adjusted P Value | |
| --- | --- | --- | --- | --- | --- | --- |
| Vector vs. VP1 | -0.3287 | -0.5757 to -0.08172 | Yes | * | 0.0288 | A-B |
| Vector vs. TBK1 | -2.407 | -2.816 to -1.997 | Yes | *** | 0.0002 | A-C |
| Vector vs. TBK1+VP1 | -4.988 | -7.613 to -2.363 | Yes | * | 0.0144 | A-D |
| VP1 vs. TBK1 | -2.078 | -2.326 to -1.830 | Yes | **** | <0.0001 | B-C |
| VP1 vs. TBK1+VP1 | -4.659 | -7.416 to -1.903 | Yes | * | 0.0181 | B-D |
| TBK1 vs. TBK1+VP1 | -2.581 | -5.580 to 0.4174 | No | ns | 0.0665 | C-D |
| Test details | Mean 1 | Mean 2 | Mean Diff. | SE of diff. | n1 | n2 |
| Vector vs. VP1 | 1 | 1.329 | -0.3287 | 0.03565 | 3 | 3 |
| Vector vs. TBK1 | 1 | 3.407 | -2.407 | 0.05907 | 3 | 3 |
| Vector vs. TBK1+VP1 | 1 | 5.988 | -4.988 | 0.3788 | 3 | 3 |
| VP1 vs. TBK1 | 1.329 | 3.407 | -2.078 | 0.03574 | 3 | 3 |
| VP1 vs. TBK1+VP1 | 1.329 | 5.988 | -4.659 | 0.3978 | 3 | 3 |
| TBK1 vs. TBK1+VP1 | 3.407 | 5.988 | -2.581 | 0.4328 | 3 | 3 |

| Tukey's multiple comparisons test | Mean Diff. | 95.00% CI of diff. | Below threshold? | Summary | Adjusted P Value | |
| --- | --- | --- | --- | --- | --- | --- |
| Vector vs. VP1 | -0.7404 | -1.039 to -0.4416 | Yes | ** | 0.0087 | A-B |
| Vector vs. MAVS | -143.1 | -271.8 to -14.38 | Yes | * | 0.0408 | A-C |
| Vector vs. MAVS+VP1 | -81.17 | -115.8 to -46.56 | Yes | ** | 0.0097 | A-D |
| VP1 vs. MAVS | -142.3 | -271.0 to -13.62 | Yes | * | 0.0412 | B-C |
| VP1 vs. MAVS+VP1 | -80.43 | -115.3 to -45.53 | Yes | ** | 0.01 | B-D |
| MAVS vs. MAVS+VP1 | 61.9 | -74.05 to 197.8 | No | ns | 0.2072 | C-D |
| Test details | Mean 1 | Mean 2 | Mean Diff. | SE of diff. | n1 | n2 |
| Vector vs. VP1 | 1 | 1.74 | -0.7404 | 0.04312 | 3 | 3 |
| Vector vs. MAVS | 1 | 144.1 | -143.1 | 18.57 | 3 | 3 |
| Vector vs. MAVS+VP1 | 1 | 82.17 | -81.17 | 4.995 | 3 | 3 |
| VP1 vs. MAVS | 1.74 | 144.1 | -142.3 | 18.58 | 3 | 3 |
| VP1 vs. MAVS+VP1 | 1.74 | 82.17 | -80.43 | 5.038 | 3 | 3 |
| MAVS vs. MAVS+VP1 | 144.1 | 82.17 | 61.9 | 19.62 | 3 | 3 |

Fig.S2. All Western blot raw images.


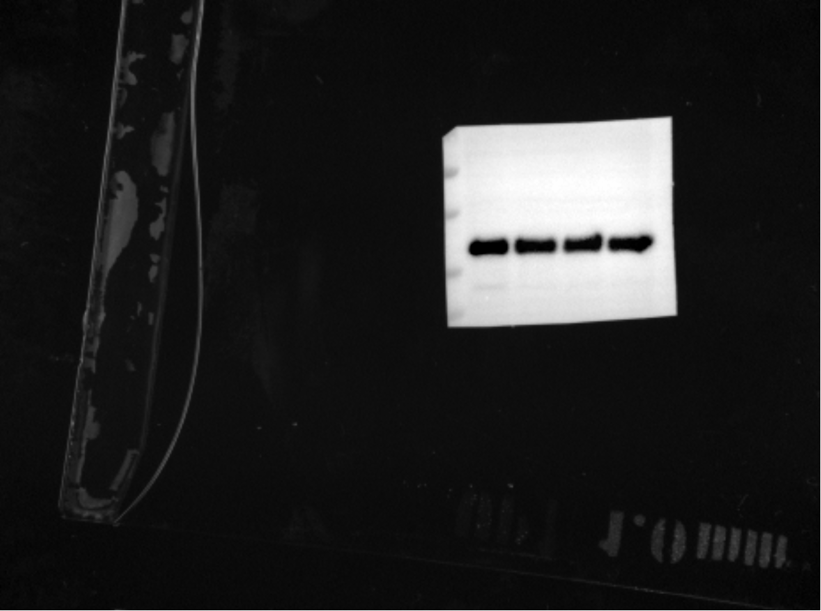


Fig.1C-GAPDH


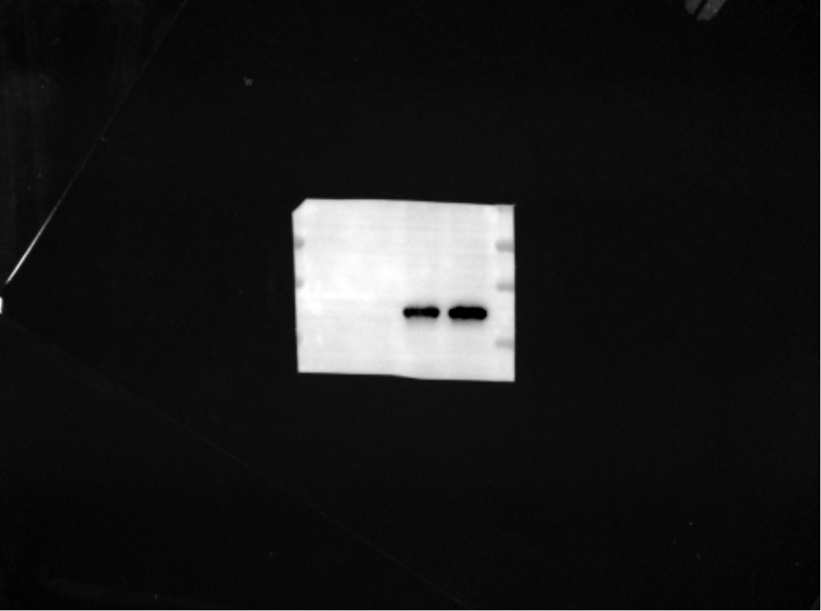


Fig.1C-HA


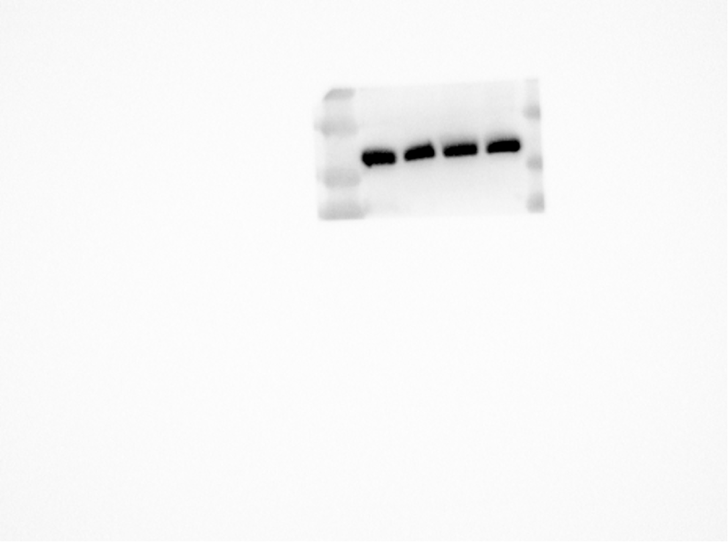


Fig.1D-GAPDH


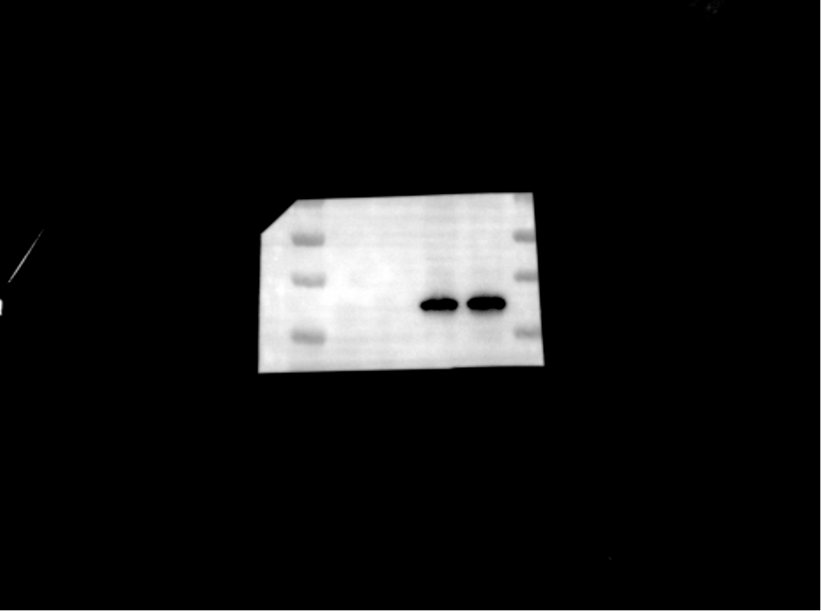


Fig.1D-HA


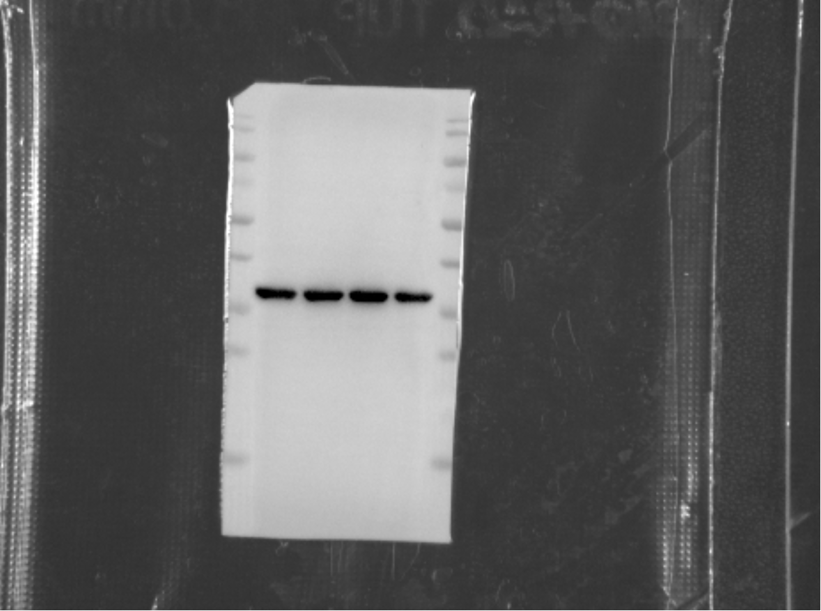


Fig.2A-GAPDH


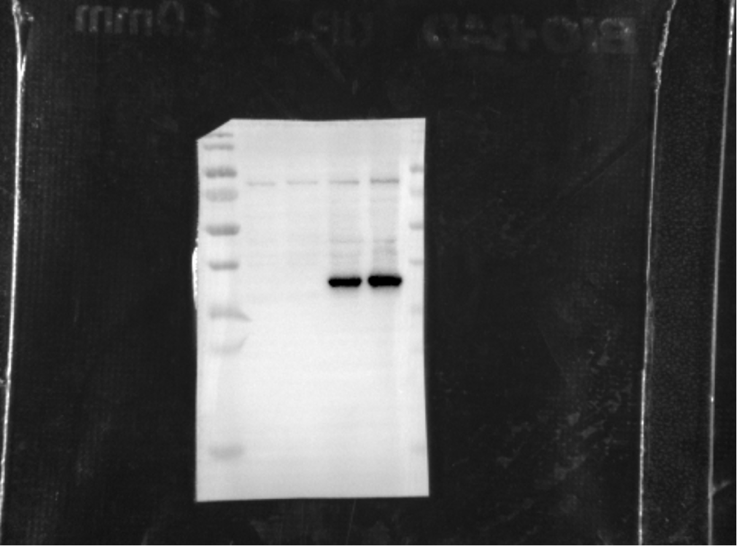


Fig.2A-HA


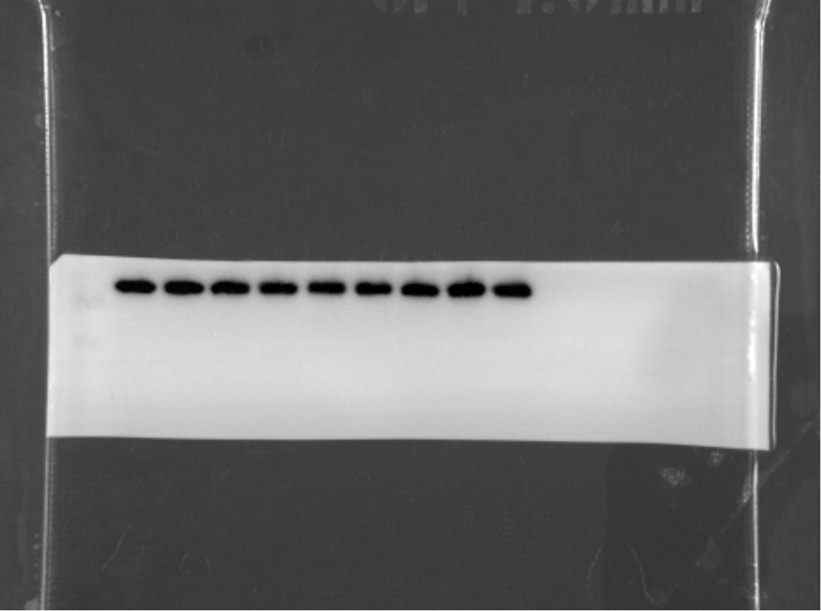


Fig.2B-GAPDH


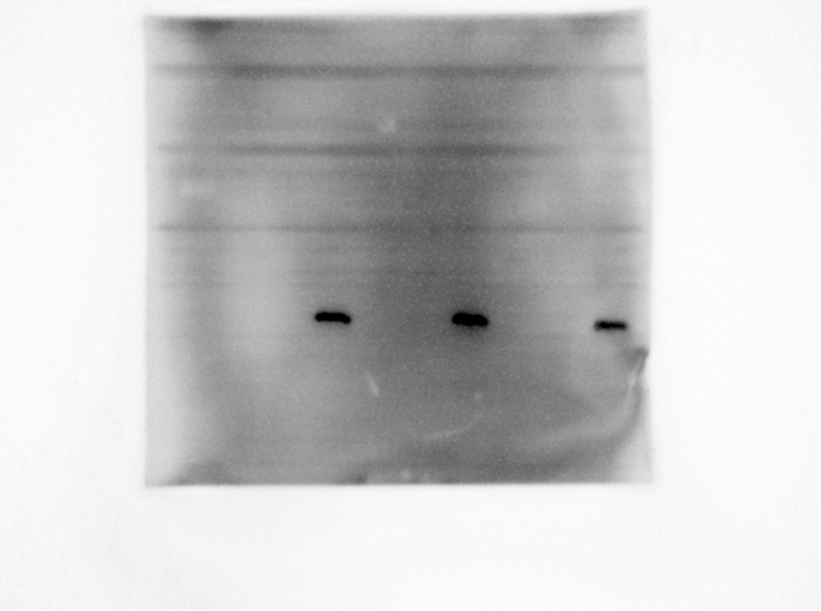


Fig.2B-HA


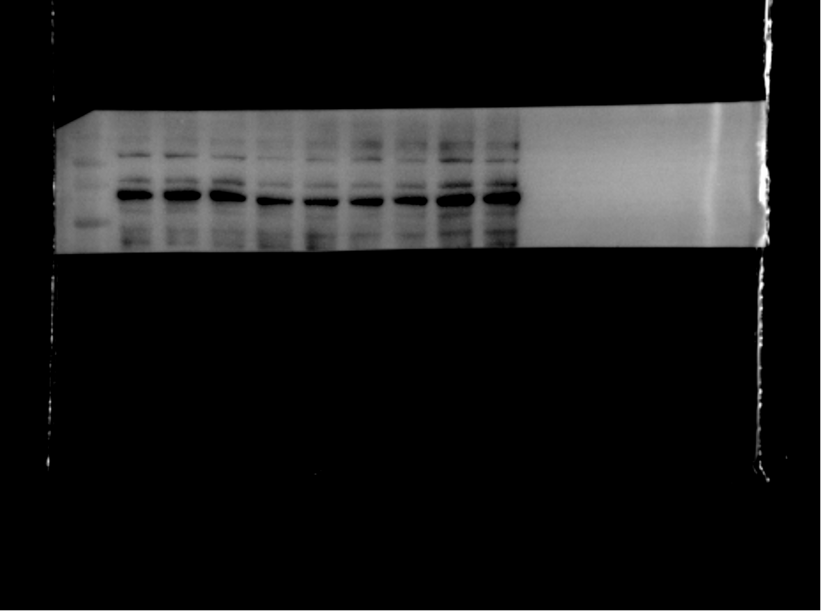


Fig.2B-NF-KB


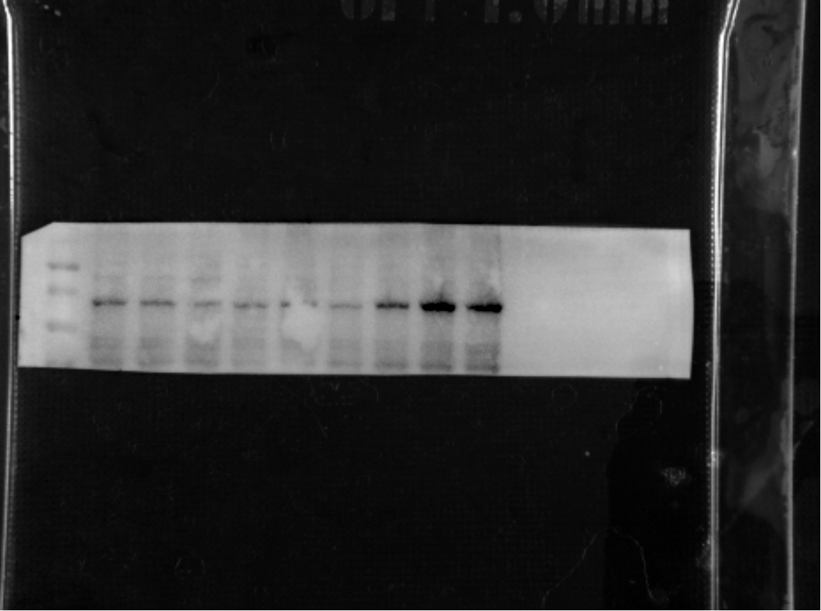


Fig.2B-p-NF-κB


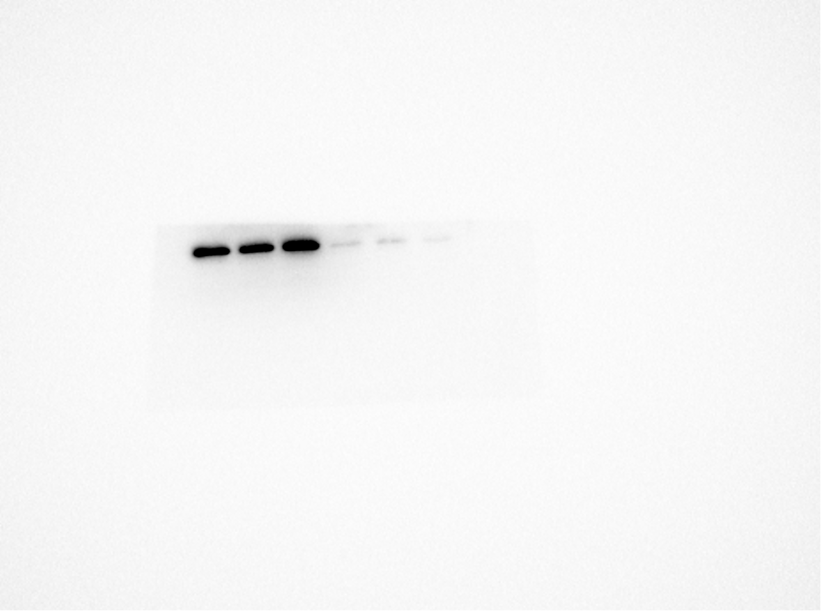


Fig.2C-cyto,nuc-GAPDH


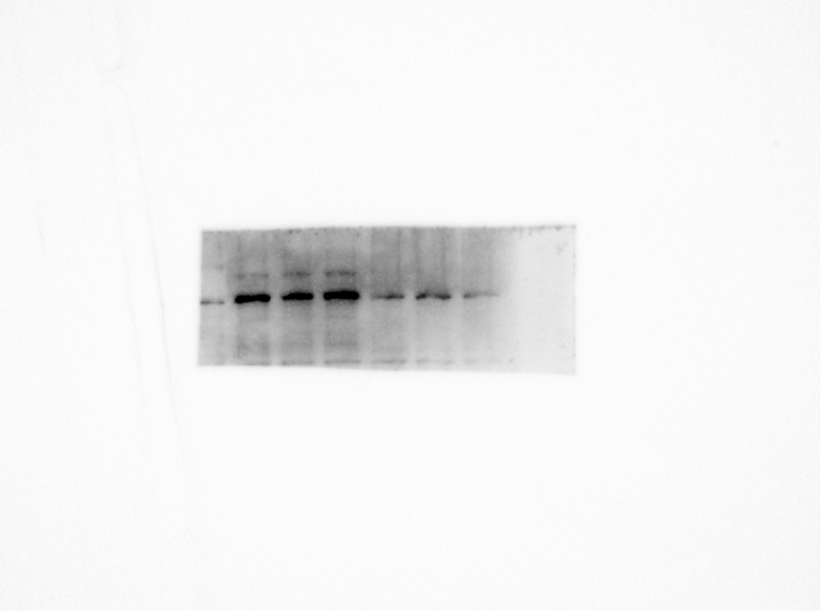


Fig.2C-cyto,nuc-NF-κB


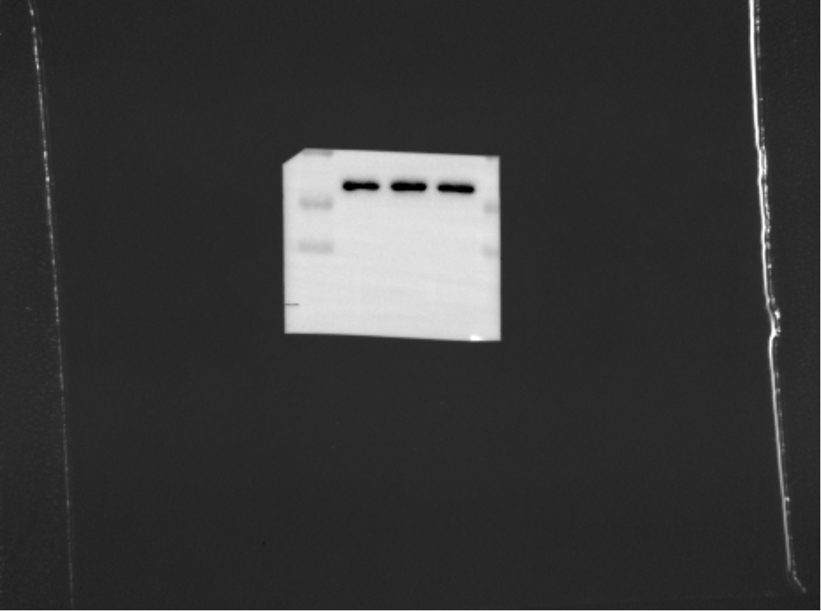


Fig.2C-wcl-GADPH


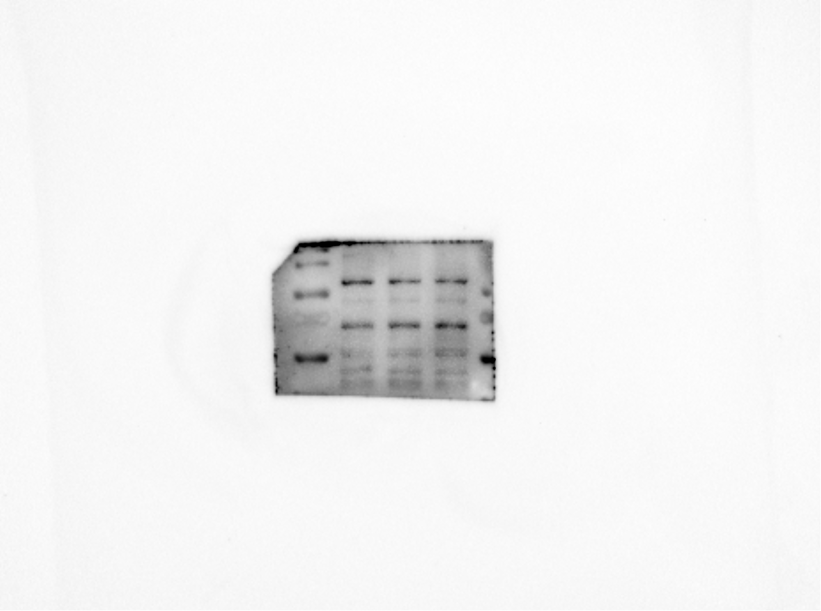


Fig.2C-wcl-NF-κB.


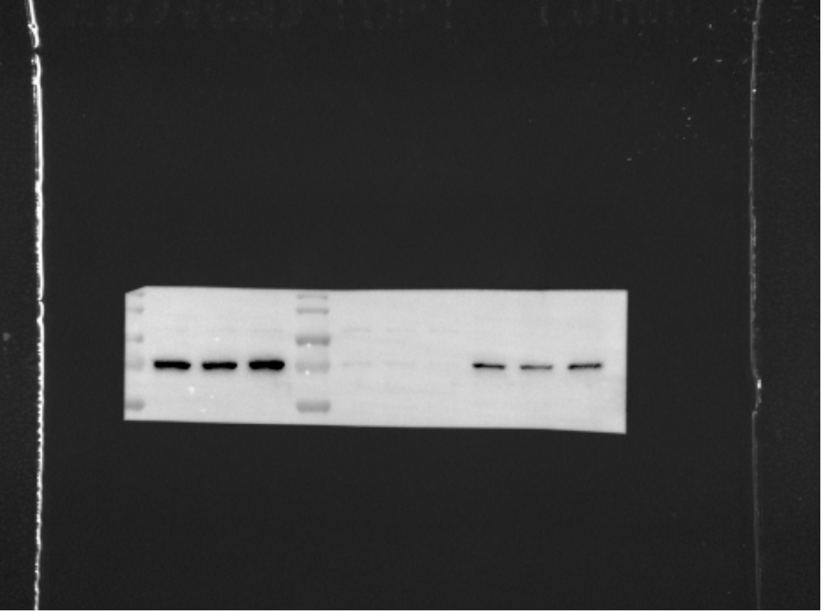


Fig.2C-WCL,cyto,nuc-lamb


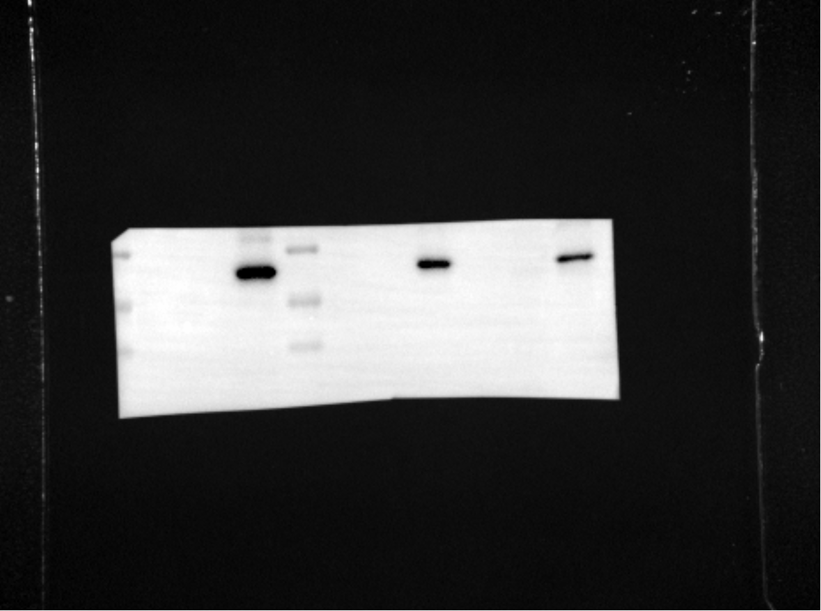


Fig.2C-WCL,cyto,nuc-VP1


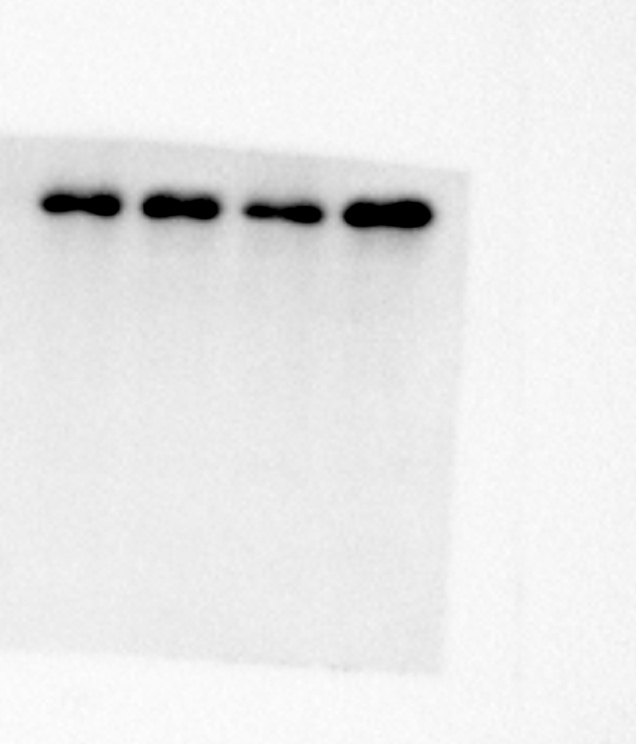


Fig.3A-GAPDH


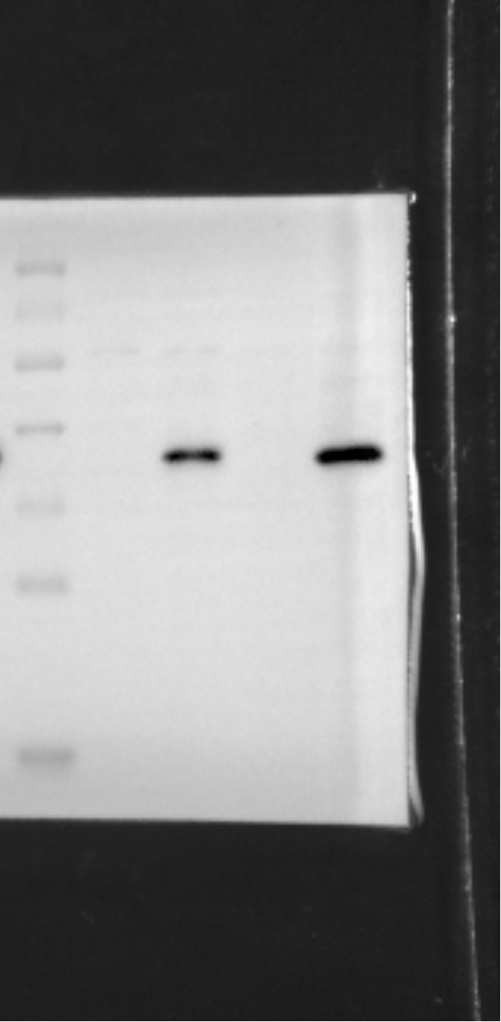


Fig.3A-HA


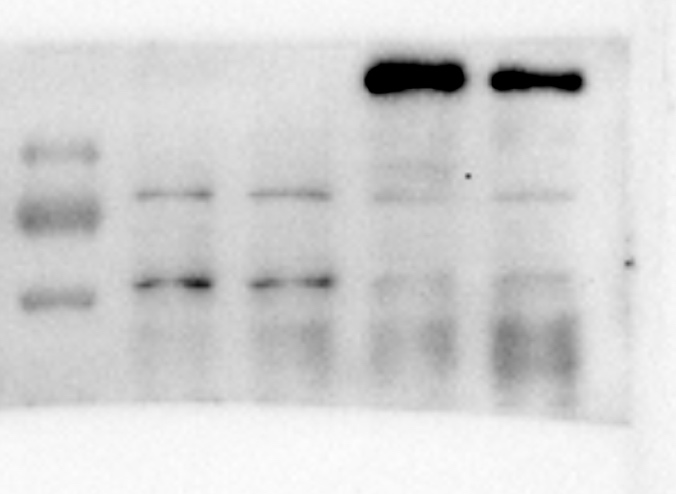


Fig.3A-MDA5


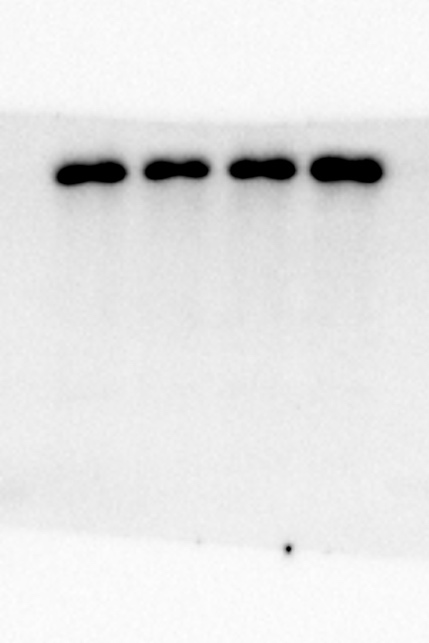


Fig.3B-GAPDH


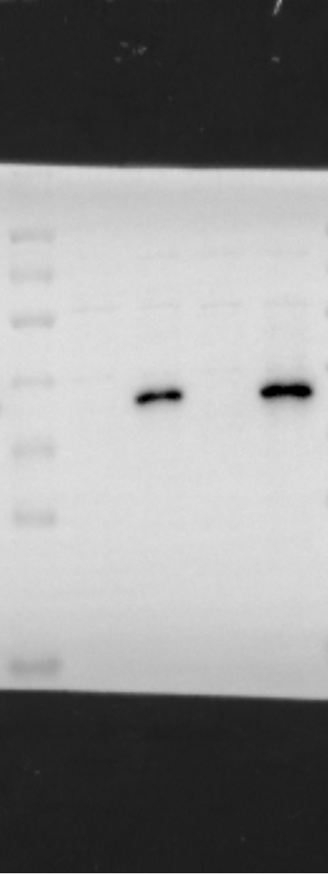


Fig.3B-HA


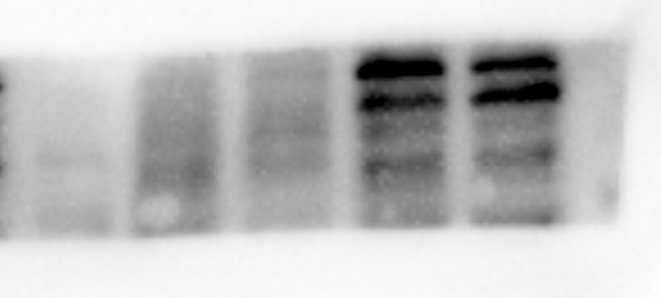


Fig.3B-RIG-I


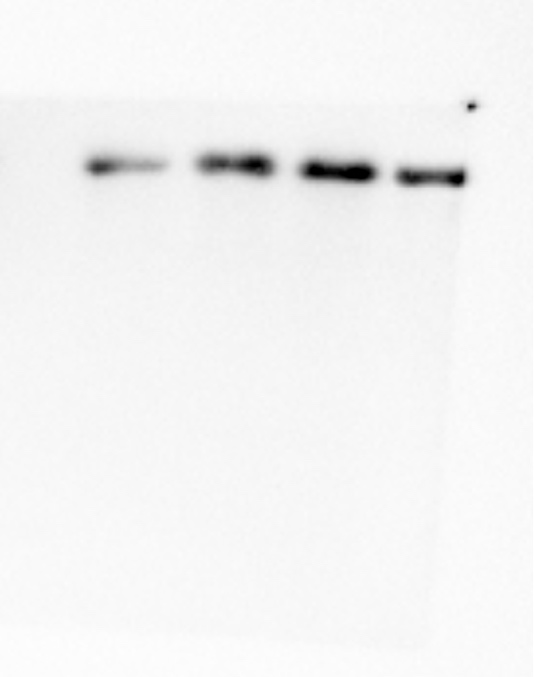


Fig.3C-GAPDH


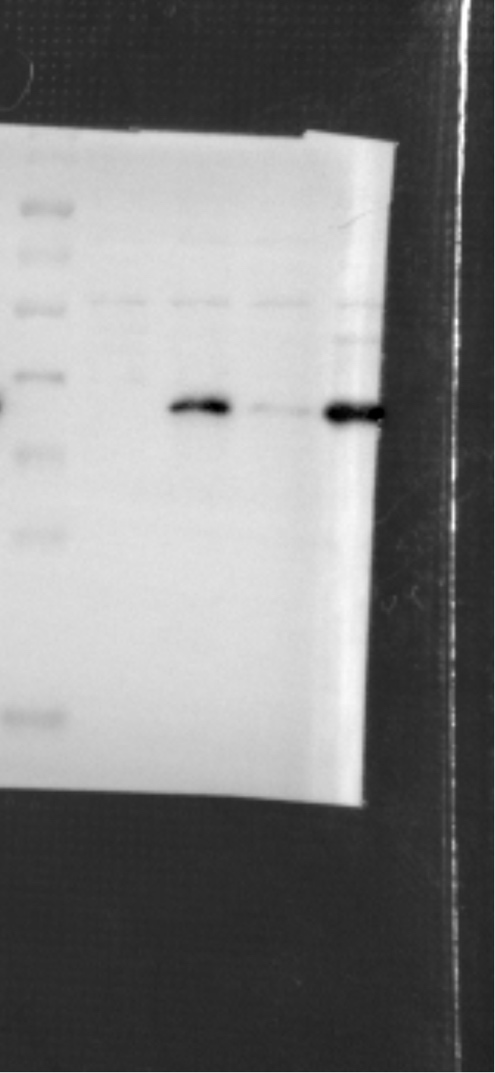


Fig.3C-HA


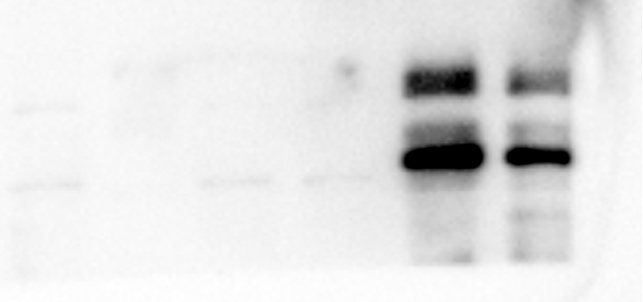


Fig.3C-MAVS


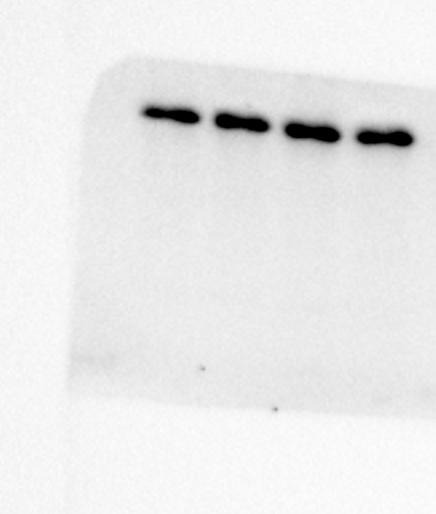


Fig.3D-GAPDH


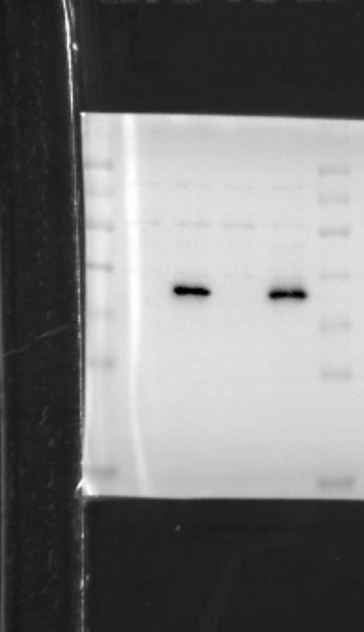


Fig.3D-HA


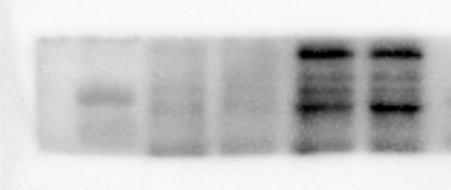


Fig.3D-TBK1


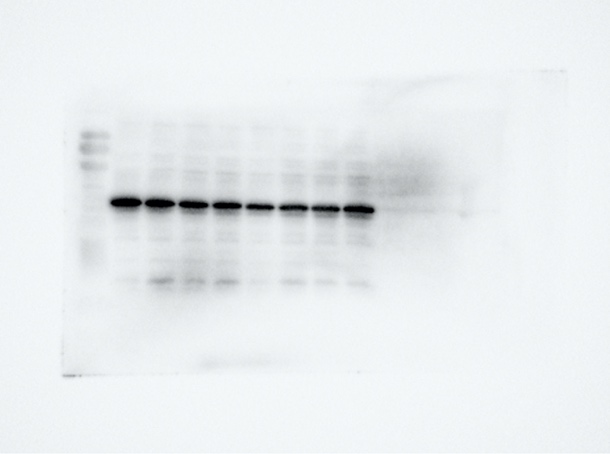


Fig.4A-GAPDH


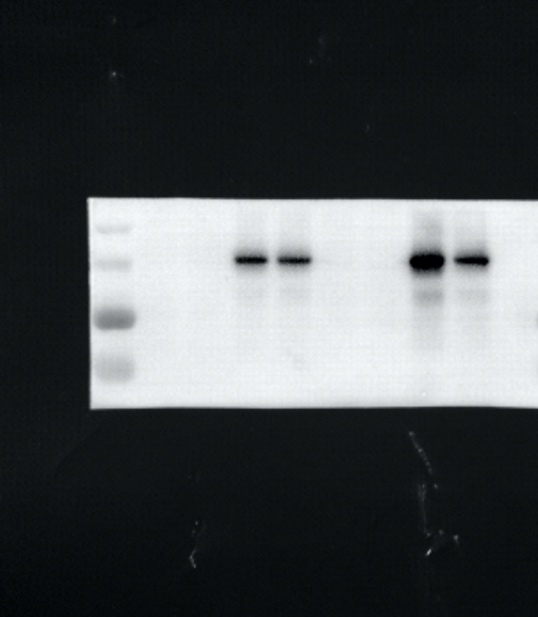


Fig.4A-MDA5


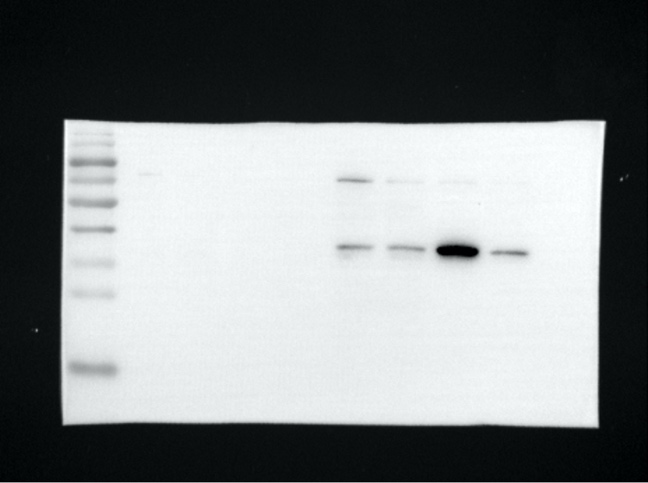


Fig.4A-VP1


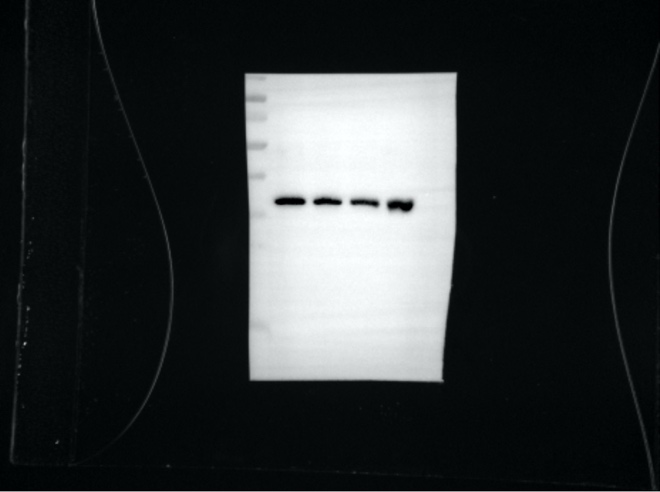


Fig.4B-GAPDH


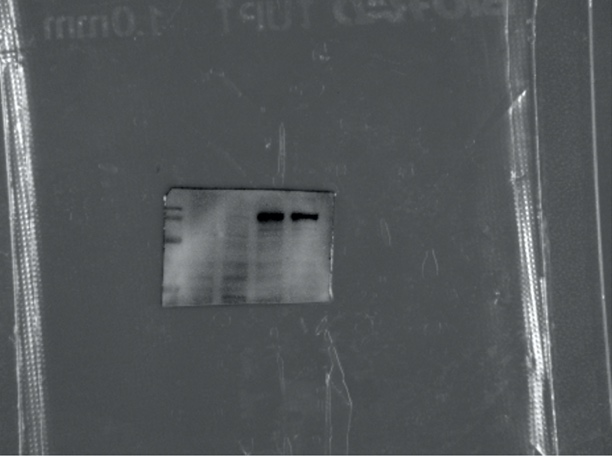


Fig.4B-MDA5


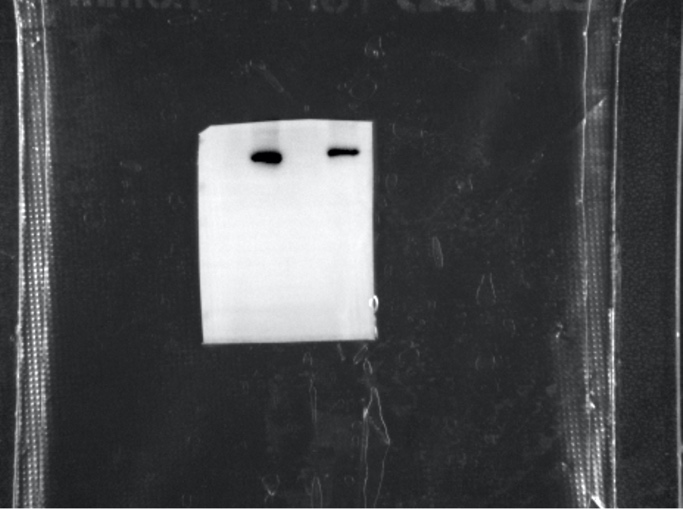


FIig.4B-VP1


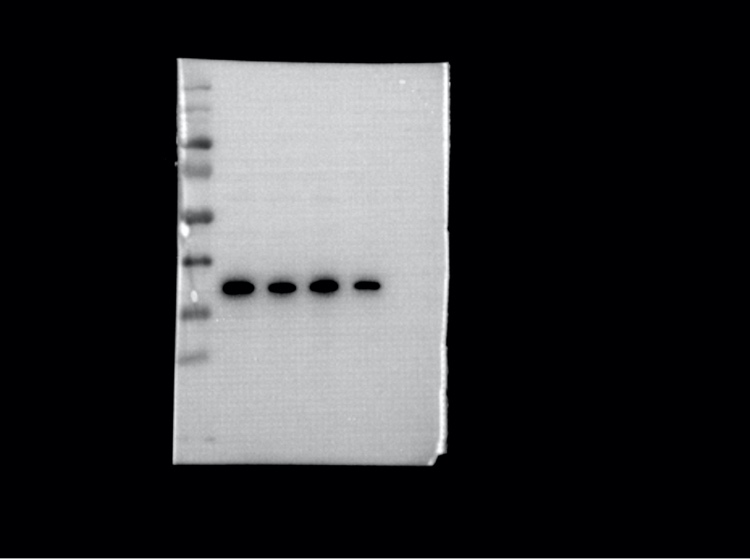


Fig.4C-input-GAPDH


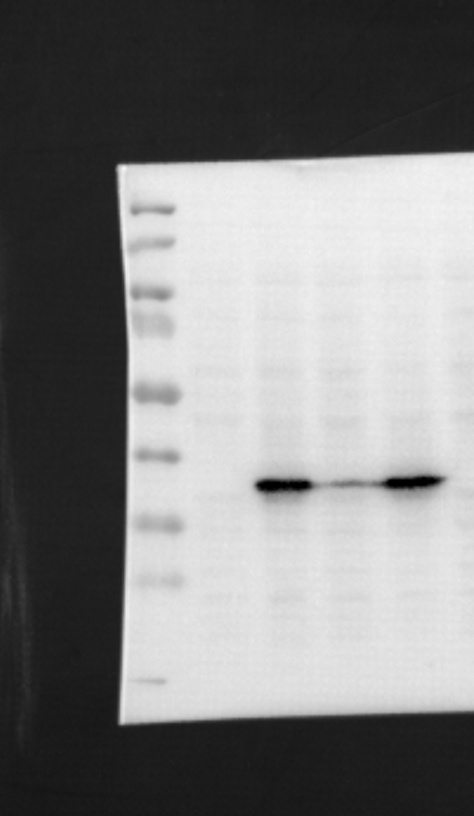


Fig.4C-input-HA


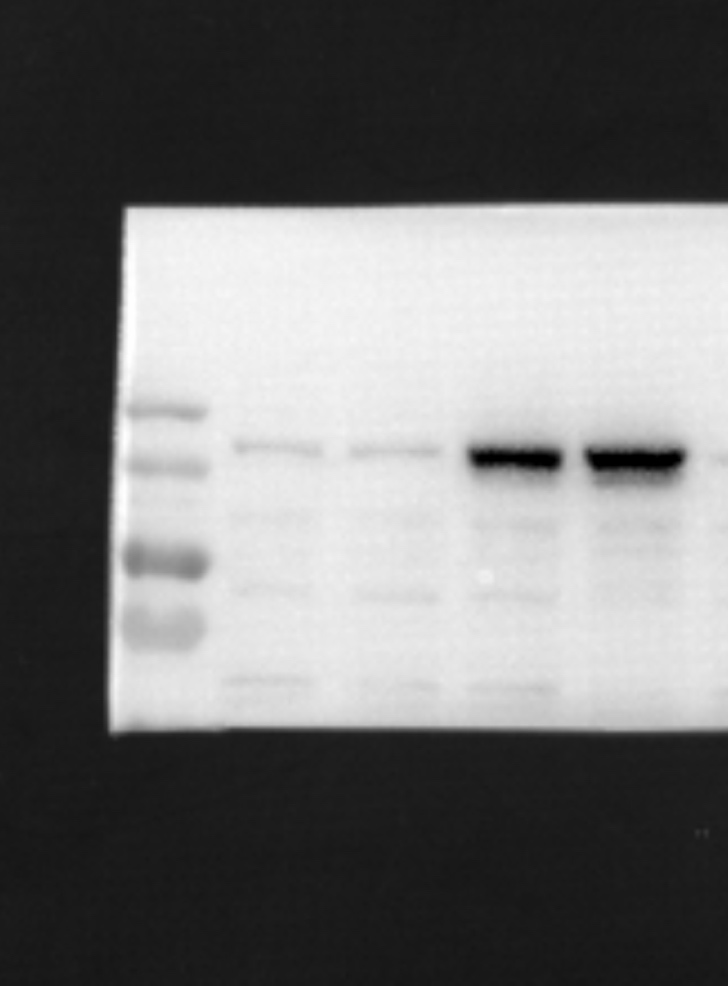


Fig.4C-input-His


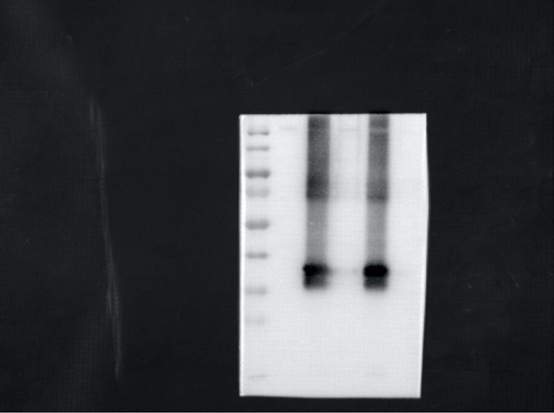


Fig.4C-IP-HA


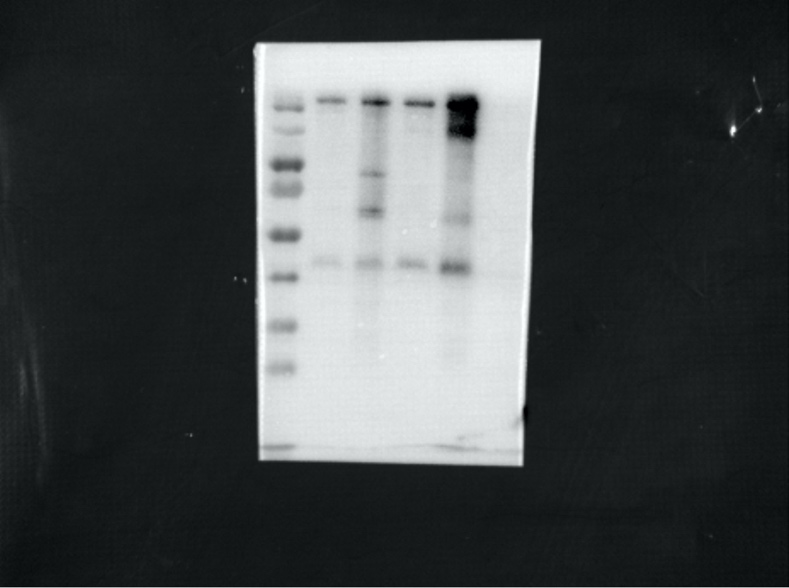


Fig.4C-IP-His


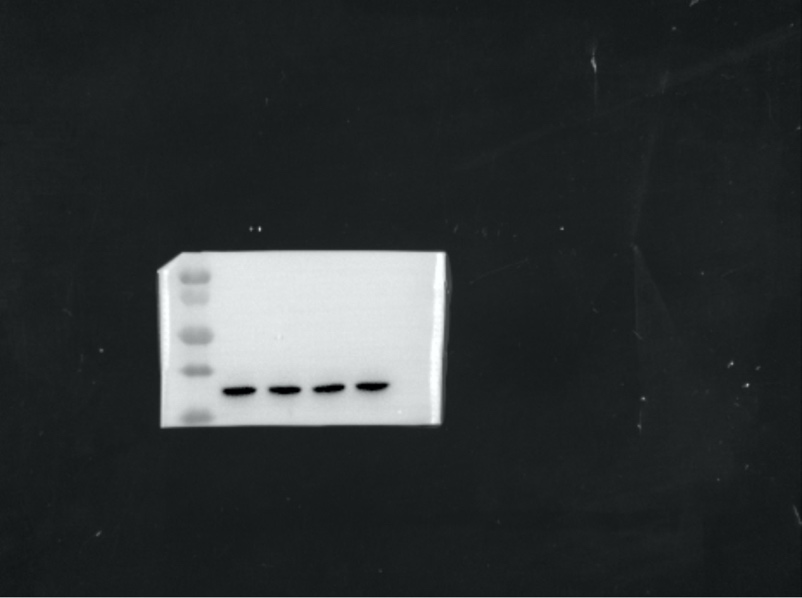


Fig.4C-Input-GADPH


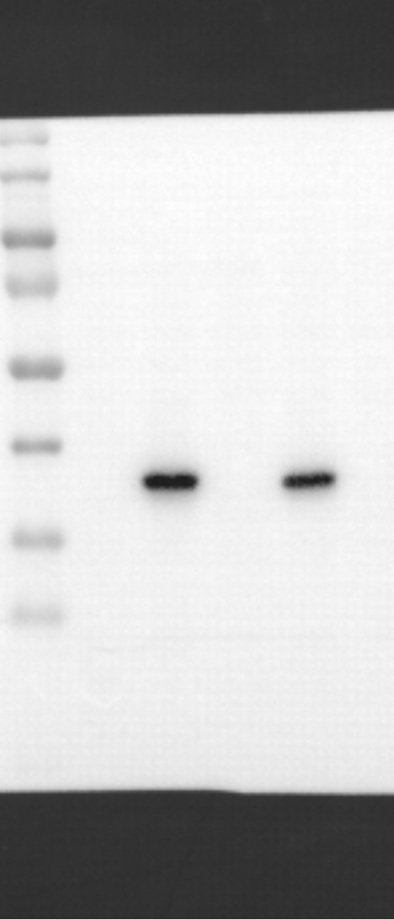


Fig.4C-Input-HA


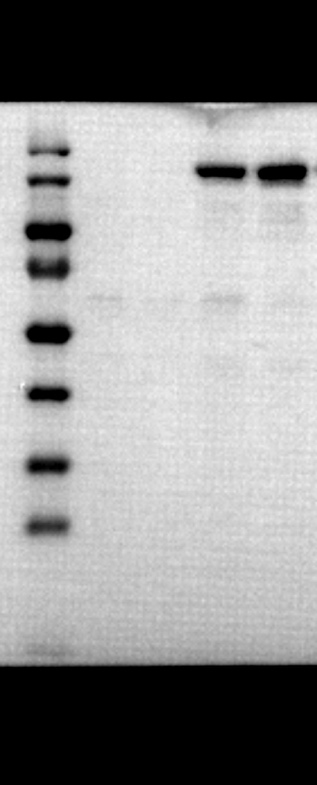


Fig.4C-Input-his


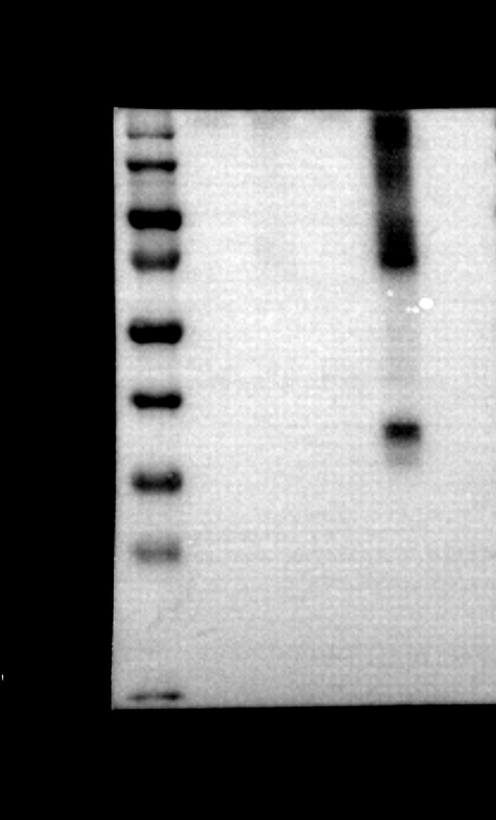


Fig.4C-IP-HA


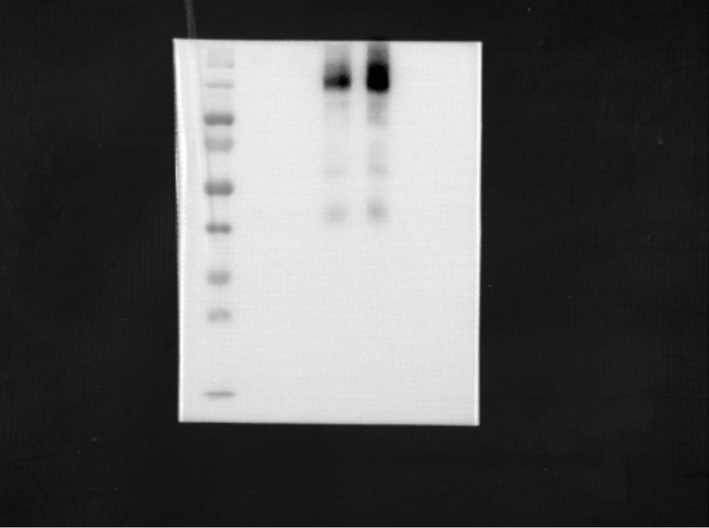


Fig.4C-IP-His


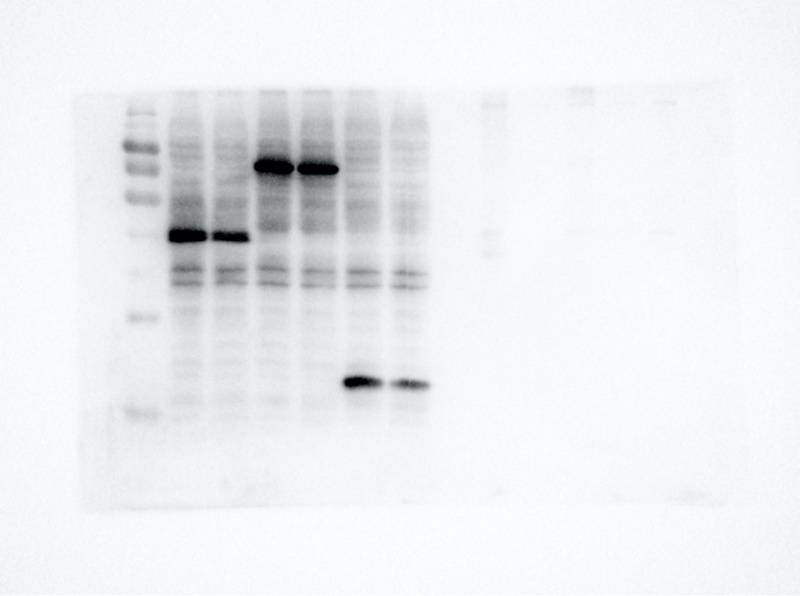


Fig.5B-input-Flag


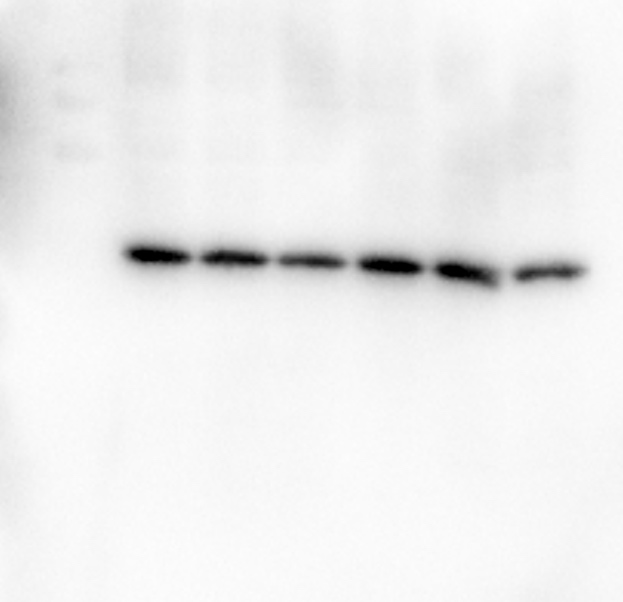


Fig.5B-input-GAPDH


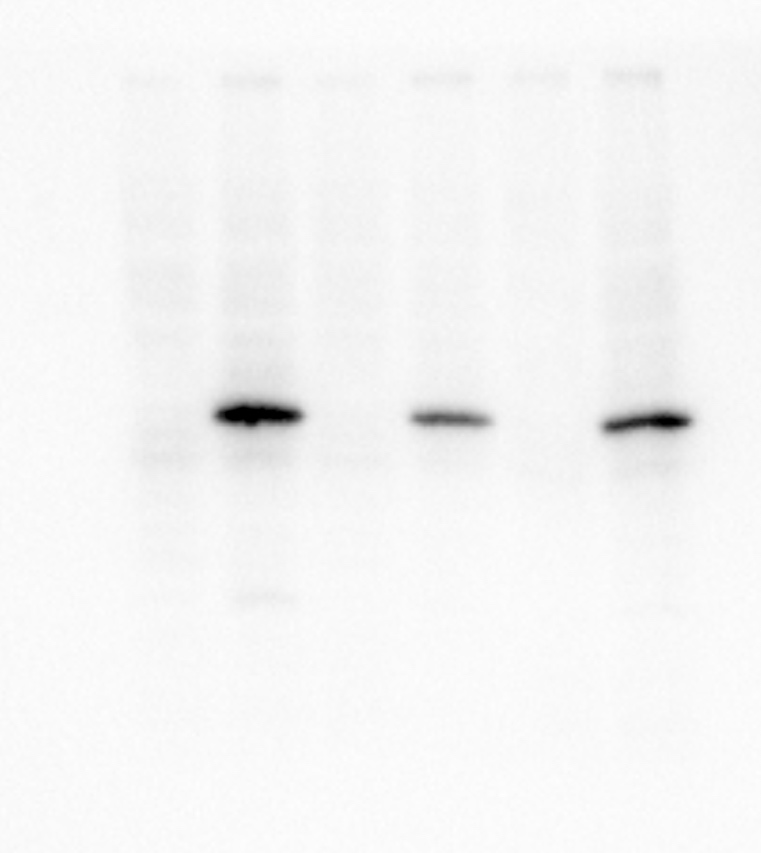


Fig.5B-input-HA


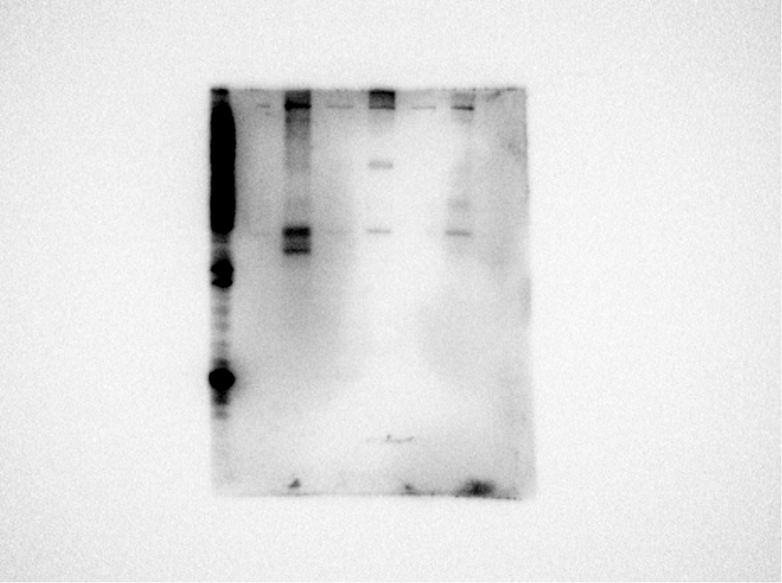


Fig.5B-ip-Flag


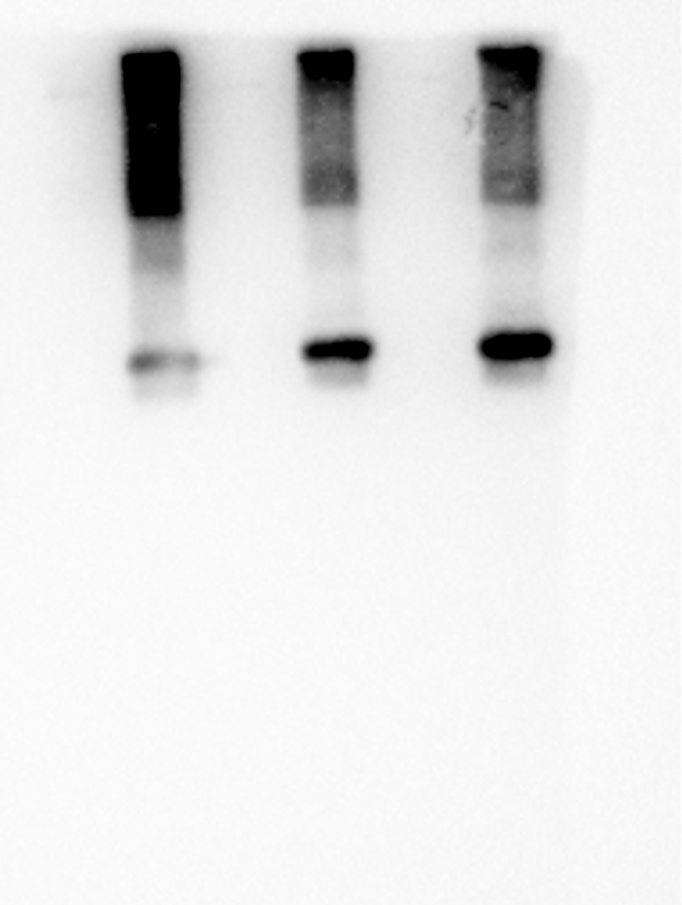


Fig.5B-ip-HA


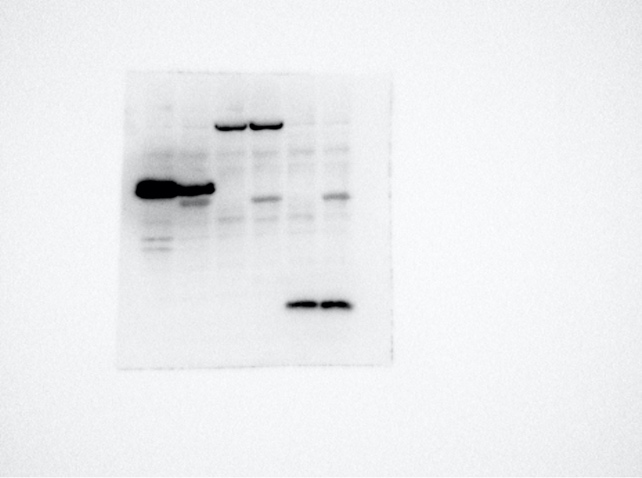


Fig.5C-input-Flag


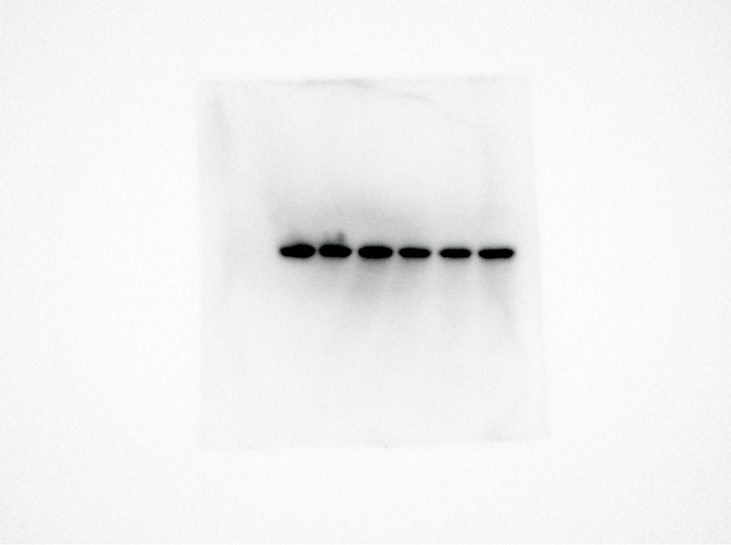


Fig.5C-input-GAPDH


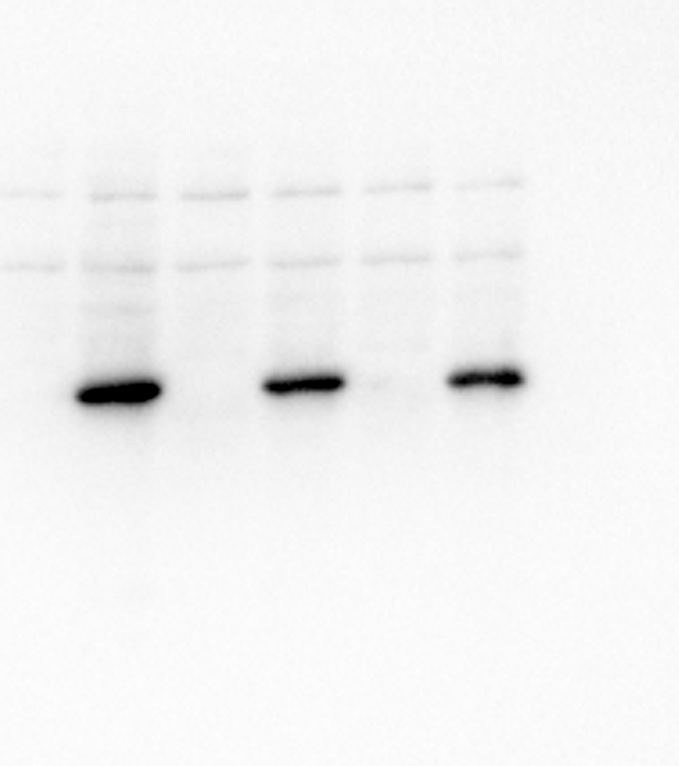


Fig.5C-input-HA


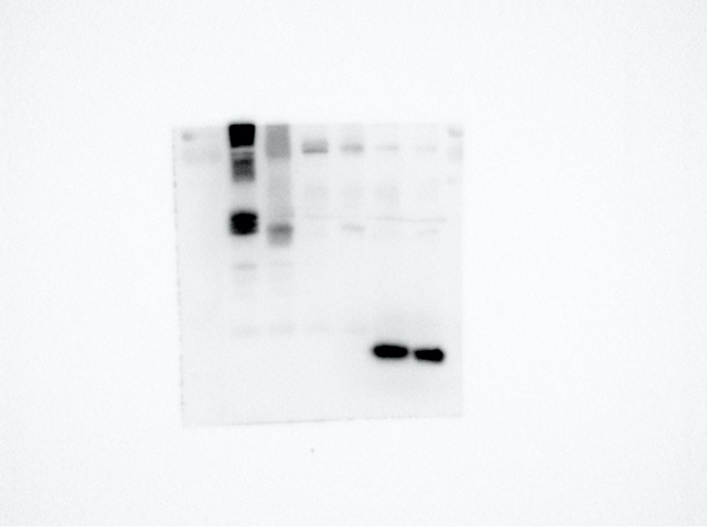


Fig.5C-ip-Flag


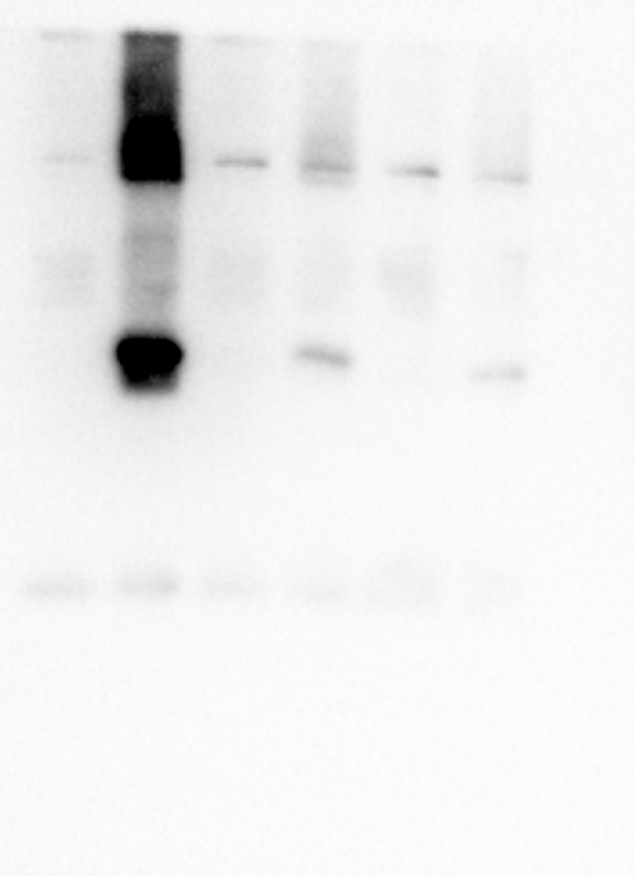


Fig.5C-ip-HA
